# Growth, Crystal Structure, Optical Limiting and Third-Order Nonlinear Optical Properties of Bis(Thiourea) Lead Dinitrate

**DOI:** 10.3390/molecules31213865

**Published:** 2026-10-26

**Authors:** Jayanthi Barasarathi, Sivaraman Shanmugam, A. Aditya Prasad, Palusamy Suppuraj, Kasi Venkatesan, Maher Fathalla, Potchanun Sripothong, Malgorzata Jeleń, Beata Morak Młodawska

**Affiliations:** 1 Faculty of Health and Life Sciences, INTI International University, Persiaran Perdana BBN, Putra Nilai, Nilai 71800, Negeri Sembilan, Malaysia; 2 Department of Chemistry, Er. Perumal Manimekalai College of Engineering, Hosur 635117, Tamilnadu, India; 3 Department of Chemistry, CMR Technical Campus, Kandlakoya, Medchal, Secunderabad 501401, Telangana, India; 4 Institute of General and Inorganic Chemistry, Academy of Sciences of the Republic of Uzbekistan, Tashkent 100170, Uzbekistan; 5 Chemistry Division, Department of H&S, CVR College of Engineering, Vastunagar, Mangalpalli Village, Rangareddy District, Ibrahimpatnam 501510, Telangana, India; 6 Department of Chemistry, Faculty of Science, Islamic University of Madinah, Madinah 42351, Saudi Arabia; 7 Faculty of Engineering and Technology, Shinawatra University, Pathum Thani 12160, Thailand; 8 Department of Organic Chemistry, Faculty of Pharmaceutical Sciences, The Medical University of Silesia, Jagiellońska 4, 41-200 Sosnowiec, Poland; manowak@sum.edu.pl (M.J.); bmlodawska@sum.edu.pl (B.M.M.)

**Keywords:** sustainable crystal growth, single-crystal, sustainable materials, manufacturing innovation, third-order nonlinear optics, optical limiting, Z-scan, Hirshfeld surface analysis, density functional theory, photoluminescence, resource efficiency, photonic materials

## Abstract

Single crystals of bis(thiourea) lead dinitrate (BTD) were successfully grown by the slow evaporation solution growth technique. Single-crystal X-ray diffraction analysis revealed that BTD crystallizes in the centrosymmetric monoclinic crystal system with the space group *P*2_1_/*c*. The phase purity and crystallinity of the grown crystals were confirmed by powder X-ray diffraction, while Fourier transform infrared (FTIR) spectroscopy verified the coordination of thiourea molecules with Pb^2+^ ions through characteristic vibrational bands. Surface morphology examined by scanning electron microscopy (SEM) showed well-developed crystal facets with only minor growth defects. Thermogravimetric and differential thermal analyses demonstrated that the crystal is thermally stable up to 188 °C. Diffuse reflectance spectroscopy (DRS) indicated excellent optical transparency in the visible region with an optical band gap of 3.27 eV, highlighting the material’s suitability for photonic applications. Photoluminescence studies exhibited an intense blue emission centered at 392 nm. Density functional theory (DFT) calculations, complemented by quantum theory of atoms in molecules (QTAIM), non-covalent interaction–reduced density gradient (NCI–RDG), electron localization function (ELF) and interaction region indicator (IRI) analyses, were performed to investigate the electronic band structure, density of states, molecular polarizability, and first-order hyperpolarizability, as well as the Pb(II) coordination environment and intermolecular interactions, providing insight into the electronic transitions governing the optical response. Hirshfeld surface and two-dimensional fingerprint analyses revealed that the crystal packing is predominantly stabilized by O···H/H···O hydrogen-bonding interactions. The third-order nonlinear optical properties were evaluated using the Z-scan technique, which demonstrated positive nonlinear refraction, self-focusing behavior, and significant nonlinear absorption, with a third-order nonlinear susceptibility of 0.9909 × 10^−13^ esu. Furthermore, the crystal exhibited efficient optical limiting with a threshold of 24.44 J cm^−2^, indicating that BTD is a promising candidate for nonlinear photonic and optical limiting applications. The potential application of these materials also highlights the importance of resource efficiency and sustainable manufacturing in future materials development.

## 1. Introduction

Nonlinear optical (NLO) materials have attracted sustained research interest owing to their indispensable role in modern photonic and optoelectronic technologies. Among the various classes of NLO materials, organic–inorganic hybrid metal coordination compounds have emerged as an important family because they effectively combine the excellent optical response and structural flexibility of organic ligands with the superior mechanical strength, thermal stability, and electronic characteristics of inorganic metal ions. The structural diversity of coordination compounds allows precise control over charge-transfer interactions, hydrogen-bonding networks, and crystal packing, which collectively influence their optical and electronic properties. Consequently, these materials have become attractive candidates for frequency conversion, optical limiting, and ultrafast photonic applications [1,2]. Recent advances in solar-driven desalination using phase-change-material storage highlight the role of functional materials in improving energy efficiency and supporting sustainable water treatment [3].

Thiourea is one of the most widely investigated ligands for the preparation of semiorganic NLO crystals because of its large molecular dipole moment, high electronic polarizability, and excellent coordination ability through the sulfur atom. Furthermore, the amino groups of thiourea readily participate in extensive intermolecular hydrogen-bonding interactions, resulting in enhanced crystal stability and favorable charge-transfer characteristics. These unique features have led to the synthesis of numerous thiourea-based coordination compounds exhibiting promising structural, optical, thermal, and nonlinear optical properties. Recent studies of metal–thiourea and related hybrid compounds, such as the mercury nitrate nonlinear optical crystal (C_5_H_12_N_2_S)Hg(NO_3_)_2_ obtained through a heteroatom coordination strategy [4], phenylthiourea-based d^10^ metal halides with markedly improved birefringence [5], and π-conjugated organic-molecule-modified metal nitrate birefringent crystals [6], further underline the importance of thiourea-based hybrid materials in the field of photo-functional materials.

Several metal–thiourea complexes have been reported, including bis(thiourea) cadmium formate [7], bis(thiourea) bismuth chloride [8], bis(thiourea) calcium chloride [9], bis(thiourea) strontium chloride [10,11], bis(thiourea) zinc acetate [12], and dichloro bis(thiourea) zinc [13,14,15,16]. Likewise, detailed crystallographic investigations have been carried out on bis(thiourea) tellurium dithiocyanate [17], bis(thiourea) tellurium halides [18], bis(thiourea) lead halides [19,20], bis(thiourea) lead nitrate [21], di-isothiocyanato bis(thiourea) chromium(II) [22], bis(acetato-κO)bis(thiourea-κS)cobalt(II) [23], bis(thiourea) gold(I) bromide [24], bis(thiourea) cadmium halides [25,26], and chloro bis(thiourea) mercury(II) chloride [27]. These studies demonstrate that the coordinated metal ion plays a crucial role in determining the crystal symmetry, hydrogen-bonding architecture, optical transparency, thermal stability, and nonlinear optical response of thiourea-based crystals.

However, the most extensively studied members of this family, such as bis(thiourea) zinc chloride [15], bis(thiourea) zinc acetate [12], bis(thiourea) cadmium chloride [28] and tris(thiourea) zinc sulfate [29], are built from closed-shell d^10^ metal ions that lack a stereochemically active lone pair and adopt tetrahedral coordination. In the present work, Pb(II) was deliberately chosen as the coordinating cation for four reasons. First, Pb^2+^ is a borderline-soft Lewis acid with a high affinity for the soft sulfur donor of thiourea, so stable Pb–S bonds form readily, while the hard oxygen donors of the nitrate anion also coordinate to give a mixed S/O coordination sphere. Second, the stereochemically active 6s^2^ lone pair of Pb(II) distorts the coordination polyhedron and increases its electronic polarizability. Third, Pb(II) adopts large and flexible coordination numbers, which allow the thiourea and nitrate ligands to assemble into extended frameworks reinforced by N–H···O hydrogen bonds. Fourth, as a heavy and highly polarizable ion, Pb^2+^ favors excited-state absorption, which is directly relevant to reverse saturable absorption and optical limiting.

These features are reflected in recent studies of Pb(II)-containing crystals. A lead mixed halide with three different coordinated anions exhibits a strong second-harmonic generation response [30], and the lead thiosemicarbazide bromides (CH_5_N_3_S)_2_Pb_5_Br_10_ and (CH_5_N_3_S)Pb_2_Br_4_, built from diverse Pb-centered units with an S-donor ligand, are also nonlinear optically active [31]. Di-aqua nitrato bis(L-valine) lead(II) nitrate single crystals show high optical transparency, efficient second- and third-order nonlinear optical responses and remarkable optical limiting behavior, highlighting the potential of Pb(II)-based coordination crystals for photonic and laser-protection applications [32]. These findings indicate that the coordination environment of Pb(II), together with ligand selection and intermolecular hydrogen bonding, plays a decisive role in tailoring the optical and nonlinear optical characteristics of lead-based crystalline materials.

Although the crystal structures of bis(thiourea) lead chloride, bromide and nitrate have been reported [19,20,21], these studies focused primarily on crystal structure determination. Detailed investigations correlating the crystal structure with the electronic structure, intermolecular interactions, photoluminescence, and third-order nonlinear optical behavior remain limited. In particular, comprehensive studies combining single-crystal X-ray diffraction, density functional theory (DFT), Hirshfeld surface analysis, photoluminescence, Z-scan measurements, and optical limiting evaluation for bis(thiourea) lead dinitrate (BTD) have not been reported. Recent investigations have further emphasized the development of hybrid coordination crystals with enhanced third-order nonlinear optical responses for advanced photonic applications [33,34,35,36,37,38,39].

In the present work, BTD crystals were successfully grown by the slow evaporation technique and systematically investigated using complementary experimental and theoretical approaches. The relationship between crystal packing, electronic structure, and nonlinear optical response has been established through SCXRD, DFT calculations, Hirshfeld surface analysis, and Z-scan measurements. Furthermore, the excellent third-order nonlinear susceptibility together with efficient reverse saturable absorption and optical limiting behavior demonstrates the potential of BTD as a candidate material for nonlinear photonic and laser-protection applications.

## 2. Experimental

### 2.1. Crystal Growth

**Figure 1:**
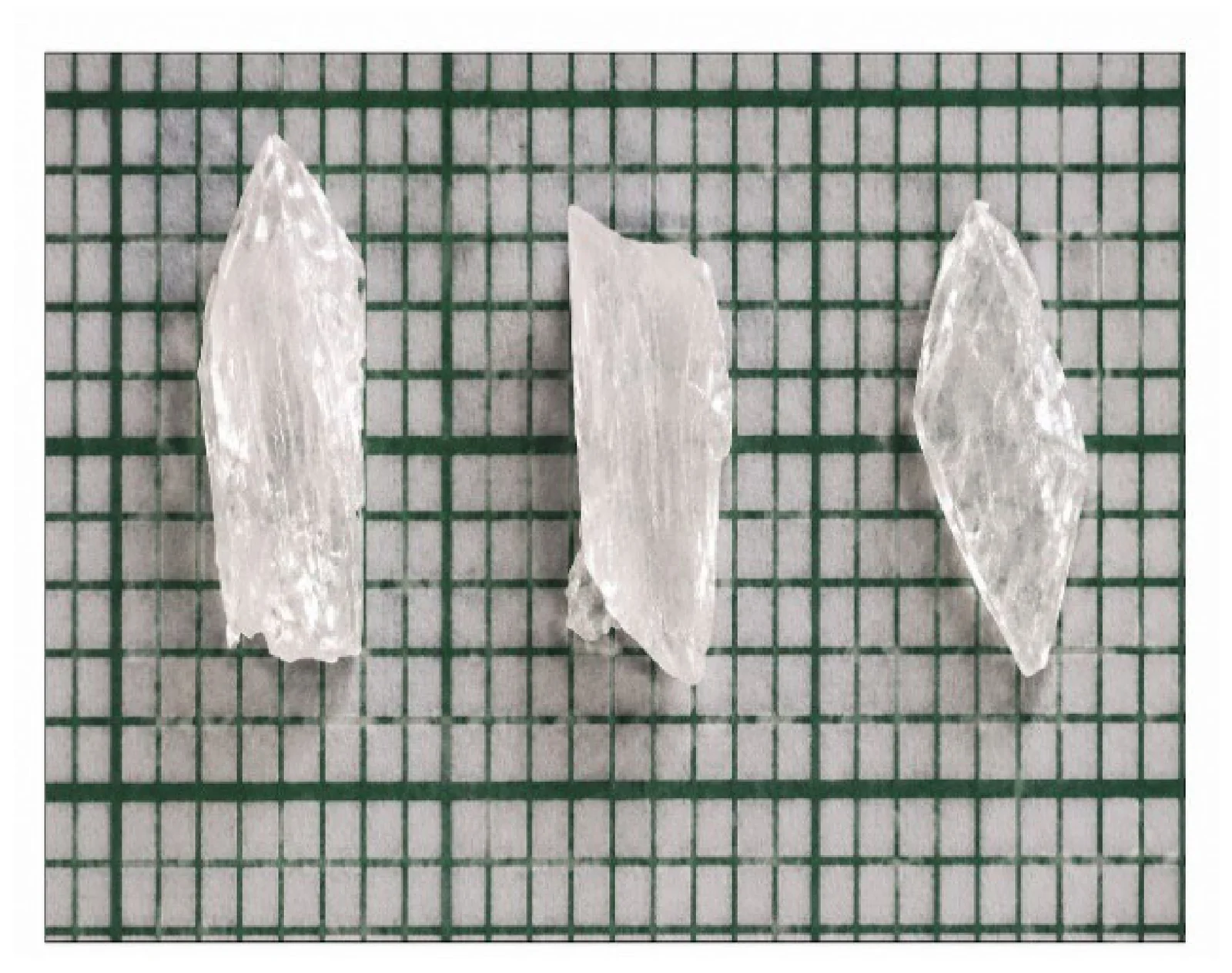
Photo images of as-grown BTD crystals.

Bis(thiourea) lead dinitrate (BTD) crystals were grown by the slow evaporation solution growth technique. Lead(II) nitrate, Pb(NO_3_)_2_ (purity-99.0%, Sigma Aldrich, Bangalore, India), and thiourea, CS(NH_2)2_ (purity-99.0%, Sigma Aldrich, Bangalore, India), were dissolved in deionized water in a stoichiometric molar ratio of 1:2 under continuous stirring to obtain a homogeneous saturated solution. The solution was filtered, repeatedly recrystallized to improve purity, and allowed to evaporate slowly at room temperature under undisturbed conditions. Transparent, well-faceted single crystals were obtained within 10–15 days. Photographs of the as-grown BTD crystals are shown in Figure 1.

### 2.2. Characterization Techniques

Single-crystal X-ray diffraction (SCXRD) data were collected on a Kappa APEX II CCD diffractometer (Bruker AXS Inc., Madison, WI, USA) using graphite-monochromated Mo Kα radiation (λ = 0.71073 Å) for a crystal of dimensions 0.400 × 0.300 × 0.150 mm^3^. FT-IR spectra were recorded in the range of 400–4000 cm^−1^ on an AVATAR 330 FT-IR spectrometer (Thermo Nicolet Corporation, Madison, WI, USA) using the KBr pellet method. Surface morphology was examined using a JSM-5610LV scanning electron microscope (JEOL Ltd., Tokyo, Japan) operated at an accelerating voltage of 15 kV, and the elemental composition was analyzed using energy-dispersive X-ray spectroscopy (EDS) with an attached detector (JEOL JSM 5610 LV, JEOL, Tokyo, Japan). Powder X-ray diffraction (PXRD) patterns were collected on a D8 diffractometer (Bruker AXS GmbH, Karlsruhe, Germany) with Cu Kα radiation (λ = 1.54056 Å) over a 2θ range of 5–50° at a scan rate of 1° min^−1^. Thermogravimetric and differential thermal analyses (TG–DTA) were carried out on a (SDT Q600 thermal analyzer, New Castle, DE, USA) at a heating rate of 20 °C min^−1^] under a nitrogen atmosphere. UV-Vis diffuse reflectance spectra were recorded in the wavelength range of 200–800 nm on a UV-vis spectra were recorded using Shimadzu UV2600 UV-Vis spectrophotometer (Labsphere Inc., North Sutton, NH, USA), and the reflectance data were converted into absorption spectra using the Kubelka–Munk function. Photoluminescence (PL) spectra were measured at room temperature in the range of 200–800 nm using an F-7000 fluorescence spectrophotometer (Hitachi High-Technologies Corporation, Tokyo, Japan). Z-scan measurement was carried out using a Q-switched Nd:YAG laser (Holmarc Opto-Mechatronics Ltd., Kochi, India) with 5 ns pulses at a repetition rate of 10 Hz, giving second harmonic at 532 nm. The laser beam was focused by a lens of 10 cm focal length.

### 2.3. Computational Studies

Density functional theory (DFT) calculations were carried out using the Gaussian 09W package (Gaussian Inc., Wallingford, CT, USA) with the B3LYP functional and the LanL2DZ basis set to optimize the molecular geometry and evaluate the electronic properties and first-order hyperpolarizability [40]. Molecular visualization was performed using GaussView 6.0 (Semichem Inc., Shawnee Mission, KS, USA) [41]. Hirshfeld surface and two-dimensional fingerprint analyses were carried out using CrystalExplorer (University of Western Australia, Perth, Australia) [42]. The electronic band structure and density of states were calculated using Virtual NanoLab 2016 (QuantumWise A/S, Copenhagen, Denmark) based on DFT with the exchange–correlation functional. The crystal morphology was simulated using WinXMorph (University of Washington, Seattle, WA, USA) from the SCXRD CIF data [43].

Topological and electron-density analyses were subsequently performed using Multiwfn 3.8 (Beijing Kein Research Center for Natural Sciences, Beijing, China) [44], with the formatted checkpoint (*.fchk*) files generated from the Gaussian calculations used as the wavefunction input. Molecular electrostatic potential (MEP) analysis [45] and quantum theory of atoms in molecules (QTAIM) analysis [46] were employed to examine the electrostatic distribution and identify bond critical points (BCPs) and associated electron-density descriptors within the Pb coordination environment. Non-covalent interaction (NCI) analysis [47] based on the reduced density gradient (RDG) [48] was used to distinguish attractive, van der Waals and repulsive regions. Additional real-space electron-density descriptors, including the electron localization function (ELF) [49], localized orbital locator (LOL) [50], average local ionization energy (ALIE) [51,52] and interaction region indicator (IRI) [53], were calculated to examine the spatial distribution and localization of electron density. Graphical visualization of the calculated electron-density distributions and isosurfaces was performed using VMD (University of Illinois at Urbana–Champaign, Urbana, IL, USA) [54].

## 3. Results and Discussion

### 3.1. FT-IR Spectral Analysis

**Figure 2:**
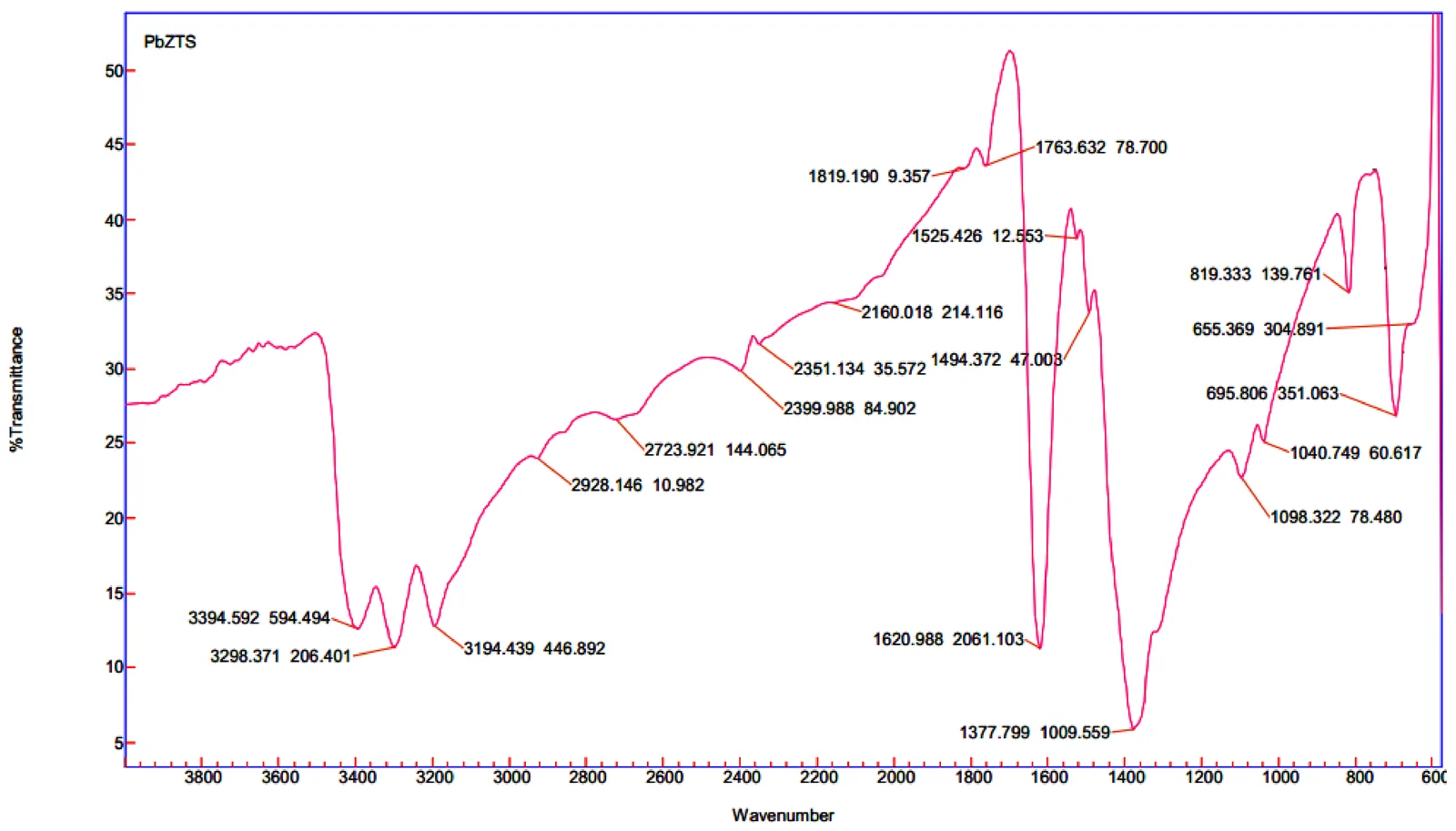
FT-IR spectrum of the as-grown bis(thiourea) lead dinitrate (BTD) crystal.

The FT-IR spectrum of the as-grown bis(thiourea) lead dinitrate (BTD) crystal recorded in the range of 400–4000 cm^−1^ is shown in Figure 2 and Table 1. The broad absorption bands observed at 3395 and 3298 cm^−1^ are assigned to the asymmetric and symmetric N–H stretching vibrations of the NH_2_ groups of thiourea, indicating the involvement of intermolecular hydrogen bonding. The weak band at 3194 cm^−1^ is also attributed to N–H stretching vibrations. The absorption band at 1621 cm^−1^ corresponds to the NH_2_ bending (δNH_2_) vibration, while the band at 1525 cm^−1^ is assigned to C–N stretching.

The strong absorption observed at 1378 cm^−1^ is attributed to the symmetric stretching vibration of the nitrate (NO_3_^−^) ion, confirming the presence of nitrate groups in the crystal structure. The characteristic bands appearing at 1098 and 1041 cm^−1^ are assigned to C=S stretching and C–N stretching vibrations of coordinated thiourea, respectively. The absorption bands at 819, 696, and 655 cm^−1^ are associated with N–C–S bending and C=S deformation modes.

### 3.2. Powder XRD Analysis

**Figure 3:**
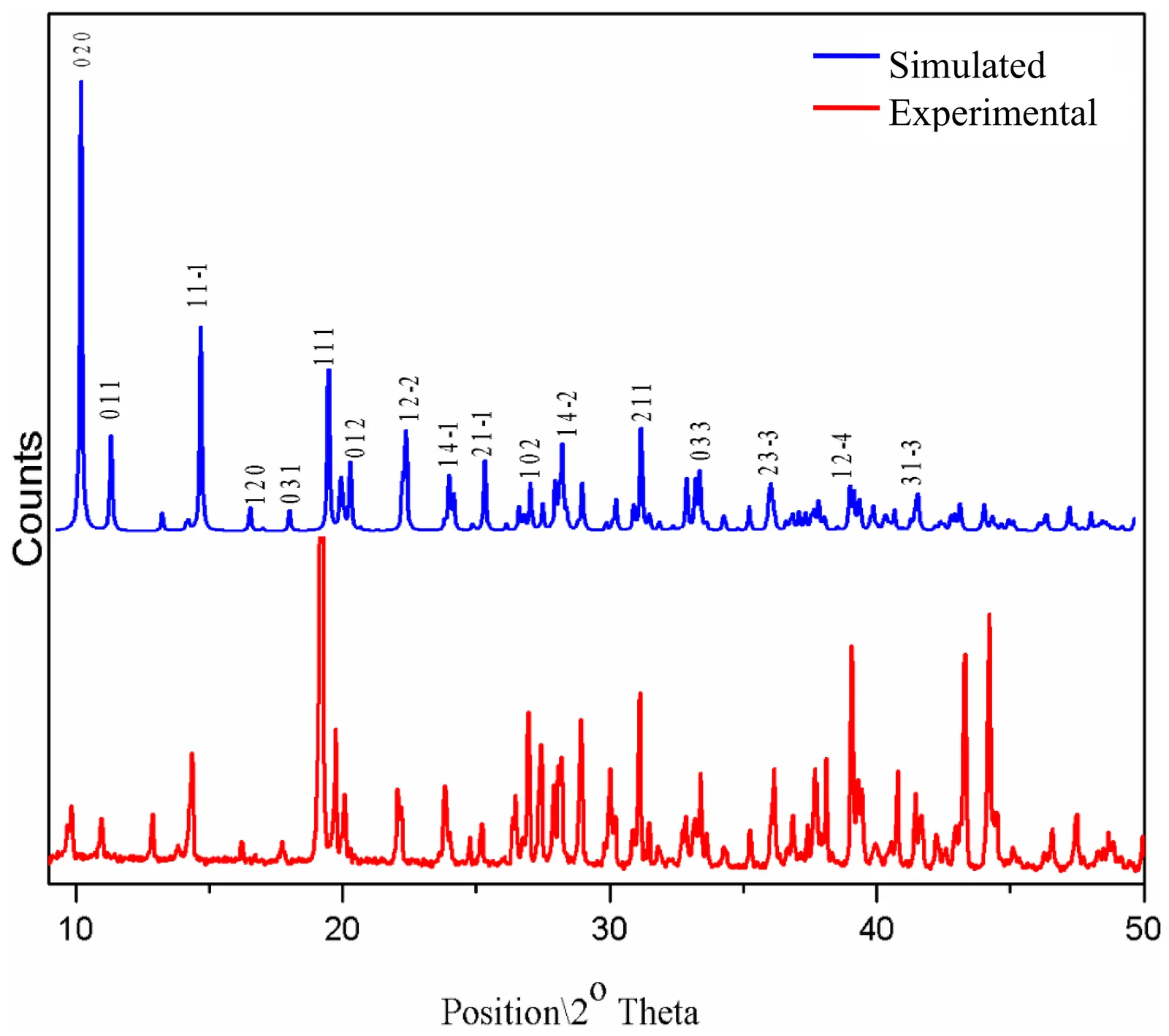
Powder XRD pattern.

The phase purity and crystallinity of the as-grown bis(thiourea) lead dinitrate (BTD) crystal were examined by powder X-ray diffraction (PXRD). The experimental PXRD pattern, together with the simulated pattern derived from the single-crystal X-ray diffraction data, is shown in Figure 3. The sharp and well-resolved diffraction peaks indicate the high crystallinity of the synthesized crystal. All observed reflections were indexed to the BTD crystal structure, and no extra peaks due to secondary phases or impurities were detected, confirming the phase purity of the sample. The experimental pattern agrees well with the simulated one, with only minor differences in relative peak intensities. These variations are attributed to preferred orientation and differences in particle size and crystallite alignment during sample preparation. The close agreement between the experimental and simulated patterns further confirms the successful growth of phase-pure BTD crystals and is consistent with the single-crystal X-ray diffraction results.

### 3.3. Single-Crystal X-Ray Diffraction Analysis

**Figure 4:**
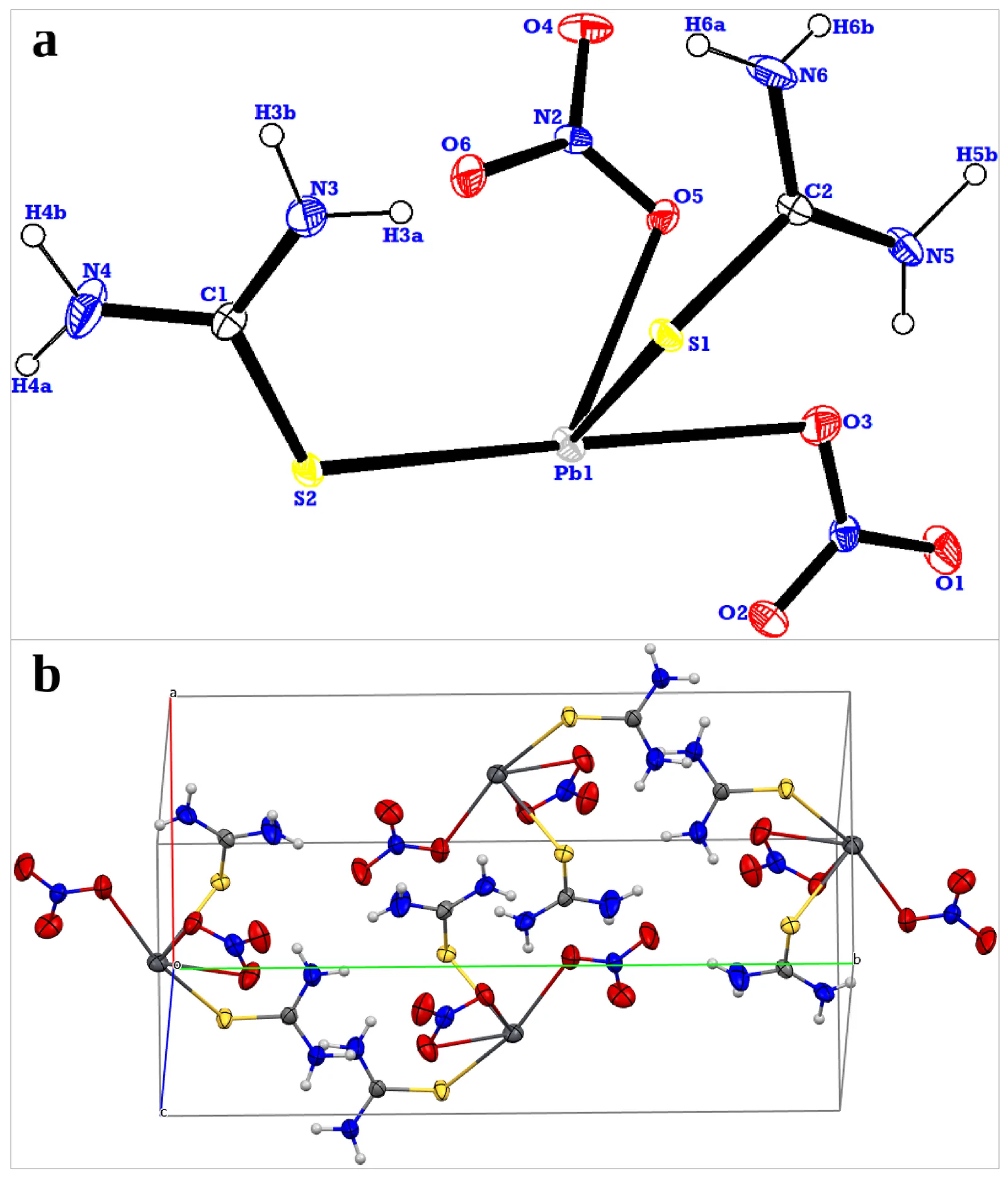
(**a**) ORTEP; (**b**) packing diagram of BTD.

The crystal structure of bis(thiourea) lead dinitrate (BTD) was determined by single-crystal X-ray diffraction (SCXRD), and the refined crystallographic parameters are summarized in Table 2. The molecular structure and crystal packing are illustrated in Figure 4. BTD crystallizes in the monoclinic crystal system with the centrosymmetric space group P2_1_/c. The refined unit-cell parameters are *a* = 7.2167(3) Å, *b* = 17.9649(8) Å, *c* = 9.4902(5) Å, *β* = 108.552(2)°, *V* = 1166.44(9) Å^3^, with *Z* = 4, confirming the formation of a well-defined crystalline phase. CCDC 1554847 contains the supplementary crystallographic data for this paper. These data can be obtained free of charge from the Cambridge Crystallographic Data Centre via https://www.ccdc.cam.ac.uk/mystructures/structuredetails/04ea4b75-0a4c-e711-9aea-005056868fc8 (accessed on 10 October 2026).

The Pb(II) ion is coordinated by two sulfur atoms from two thiourea ligands together with oxygen atoms from nitrate anions, resulting in a distorted coordination geometry around the metal center. The Pb–S bond distances vary from 2.8527(15) to 3.0506(1) Å, while the Pb–O bond distances lie in the range of 2.924(5)–3.031(5) Å. The observed S–Pb–S and C–S–Pb bond angles of 80.46(4)° and 107.21(19)°, respectively, indicate significant distortion from ideal tetrahedral geometry. This distortion is attributed to the stereochemically active 6s^2^ lone-pair electrons of the Pb(II) ion, which are known to influence the coordination environment and enhance the optical nonlinearity of lead(II) compounds [30,31].

The crystal structure is further stabilized by an extensive network of hydrogen-bonding interactions involving the amino groups of the thiourea ligands and the oxygen atoms of the nitrate anions. Strong N–H···O hydrogen bonds, including N(5)–H(5B)···O(5), N(4)–H(4A)···O(2), and N(4)–H(4B)···O(6), exhibit H···O distances of 2.02(11), 2.15(8), and 2.25(10) Å, respectively, and play a dominant role in stabilizing the molecular framework. In addition, weaker intermolecular interactions such as N–H···S and N–H···N further reinforce the three-dimensional supramolecular architecture. The combined hydrogen-bonding network contributes significantly to the structural integrity and crystal packing, which is expected to influence the thermal stability and optical properties of the material.

### 3.4. Scanning Electron Microscopy (SEM)

**Figure 5:**
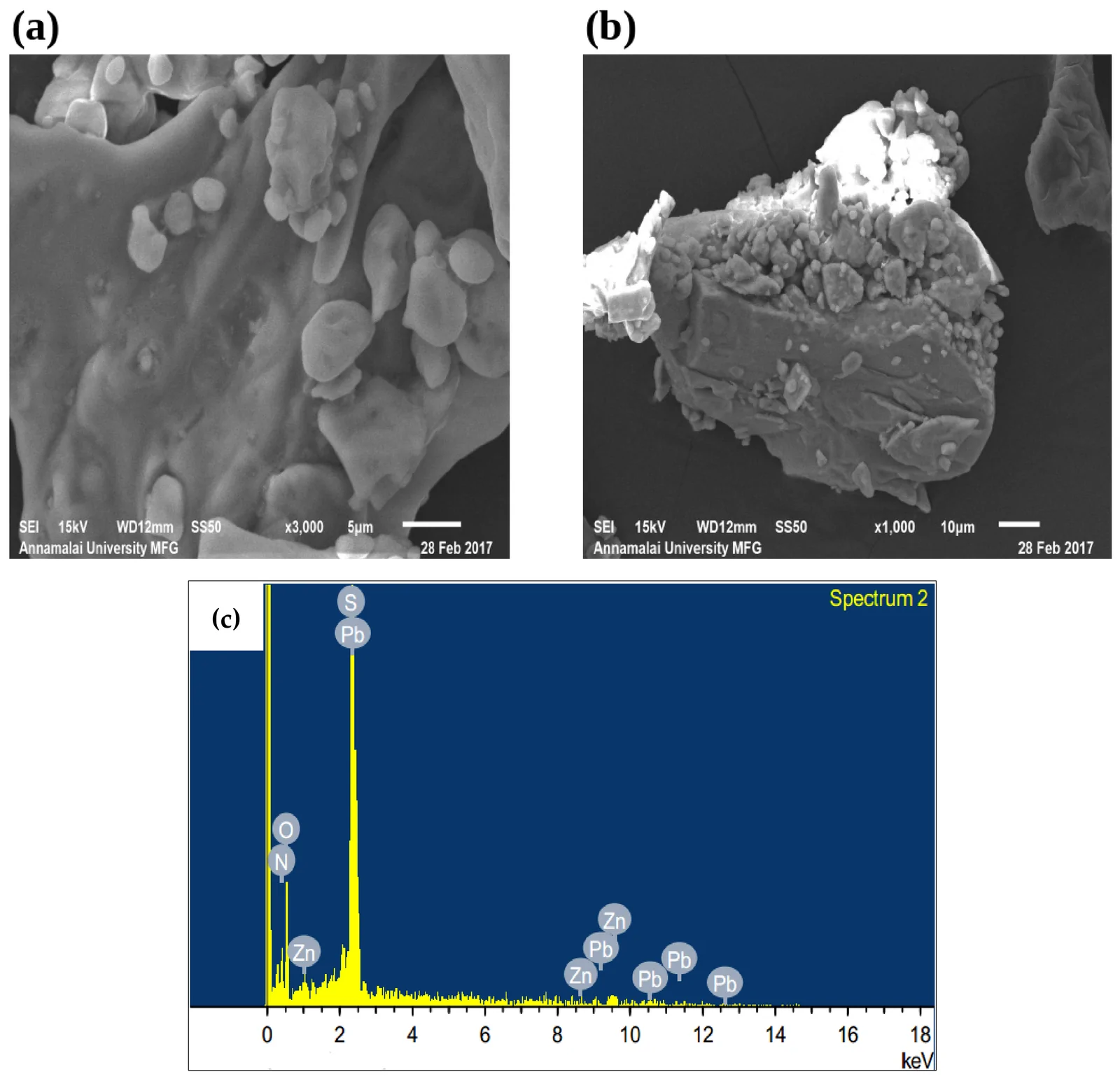
(**a**,**b**) SEM micrographs of the as-grown bis(thiourea) lead dinitrate (BTD) crystal at magnifications of ×3000 and ×1000, respectively; (**c**) EDS spectrum of BTD.

The surface morphology of the as-grown bis(thiourea) lead dinitrate (BTD) crystal was examined by SEM, and the micrographs are shown in Figure 5a,b, and the EDS spectrum is shown in Figure 5c. The images reveal a compact surface with agglomerated grains and moderate surface roughness. A few localized microvoids and surface irregularities are observed, which are attributed to the crystal growth process. No significant cracks or secondary phases are detected, indicating good crystalline quality. The EDS spectrum confirms the presence of Pb, S, O, N and C, the constituent elements of BTD (hydrogen cannot be detected by EDS).

### 3.5. Thermal Studies

**Figure 6:**
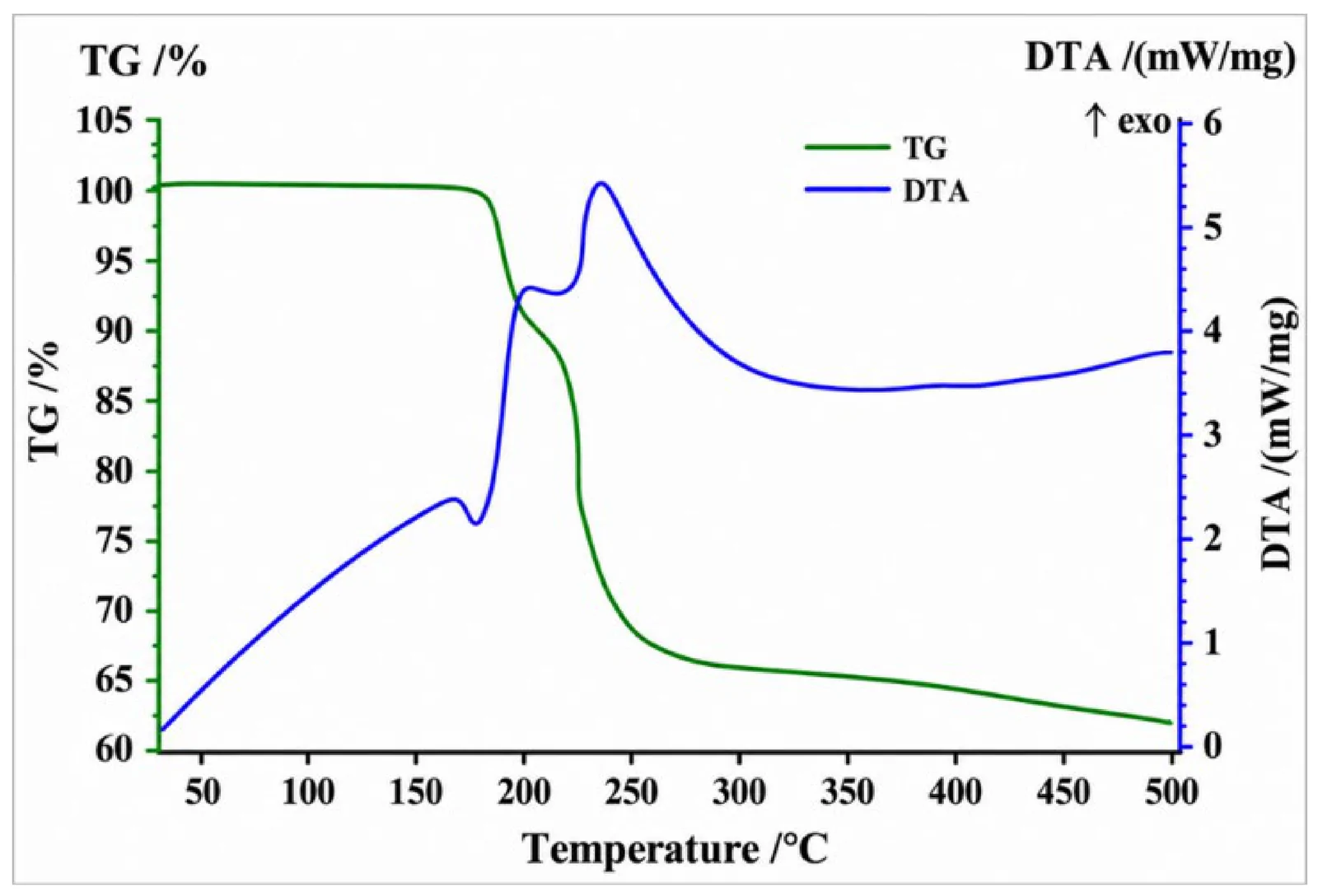
TG-DTA curve of BTD.

The thermal stability of the BTD crystal was investigated using thermogravimetric analysis (TG) and differential thermal analysis (DTA), and the corresponding curves are presented in Figure 6. The TG curve shows that the crystal remains thermally stable up to approximately 188 °C, with no significant weight loss observed below this temperature, confirming the absence of lattice or adsorbed water molecules. The onset of decomposition occurs near 188 °C, followed by a major weight loss between 188 and 246 °C, corresponding to the release of volatile constituents and decomposition of the organic moiety. The total weight loss in this temperature range is approximately 29.25%. Beyond 250 °C, the decomposition proceeds gradually, leaving a stable residual mass at higher temperatures.

The DTA curve exhibits a sharp endothermic peak at 188 °C, which is assigned to the melting of the BTD crystal. The sharpness and intensity of this peak indicate the high crystallinity and good phase purity of the grown crystal. A second broad endothermic event centered at approximately 228 °C is attributed to the thermal decomposition of the molten compound, consistent with the significant mass loss observed in the TG curve. The thermal analysis demonstrates that the BTD crystal is thermally stable up to 188 °C, making it suitable for applications operating below this temperature.

### 3.6. UV–Vis–NIR Diffuse Reflectance Studies

**Figure 7:**
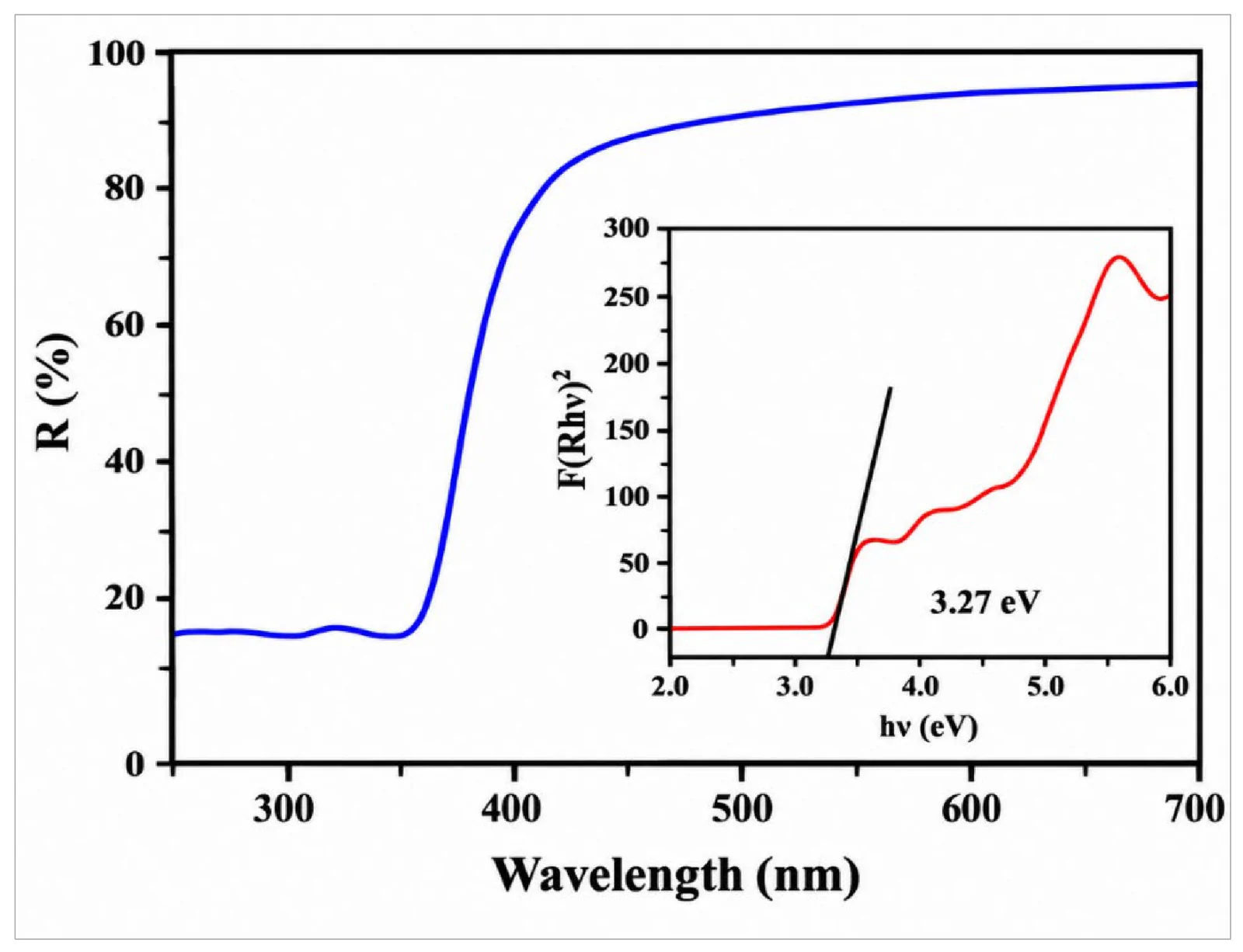
UV-vis spectrum of BTD (Tauc plot is given as an inset).

The UV–Vis–NIR diffuse reflectance spectrum of the BTD crystal is shown in Figure 7. The crystal exhibits high reflectance in the visible region with a sharp absorption edge at approximately 359 nm, indicating low optical absorption and good optical transparency.

The optical band gap was estimated using the Kubelka–Munk function [55],

$$F(R)=\frac{{\left(1-R\right)}^{2}}{2R}=\frac{\alpha }{S}=\frac{Ac}{S}$$

where *R* is the reflectance, *F(R)* is the Kubelka–Munk function, α is the absorption coefficient, *S* is the scattering coefficient, *A* is the absorbance, and *c* is the concentration of the absorbing species. The direct optical band gap was determined from the *Tauc* plot of ${\left[F(R)h\nu \right]}^{2}$ versus hν (inset of Figure 7). Extrapolation of the linear region gives a band gap energy of 3.27 eV, confirming the wide band gap and excellent optical transparency of the crystal, making it a promising material for nonlinear optical and optoelectronic applications.

### 3.7. Photoluminescence Studies

**Figure 8:**
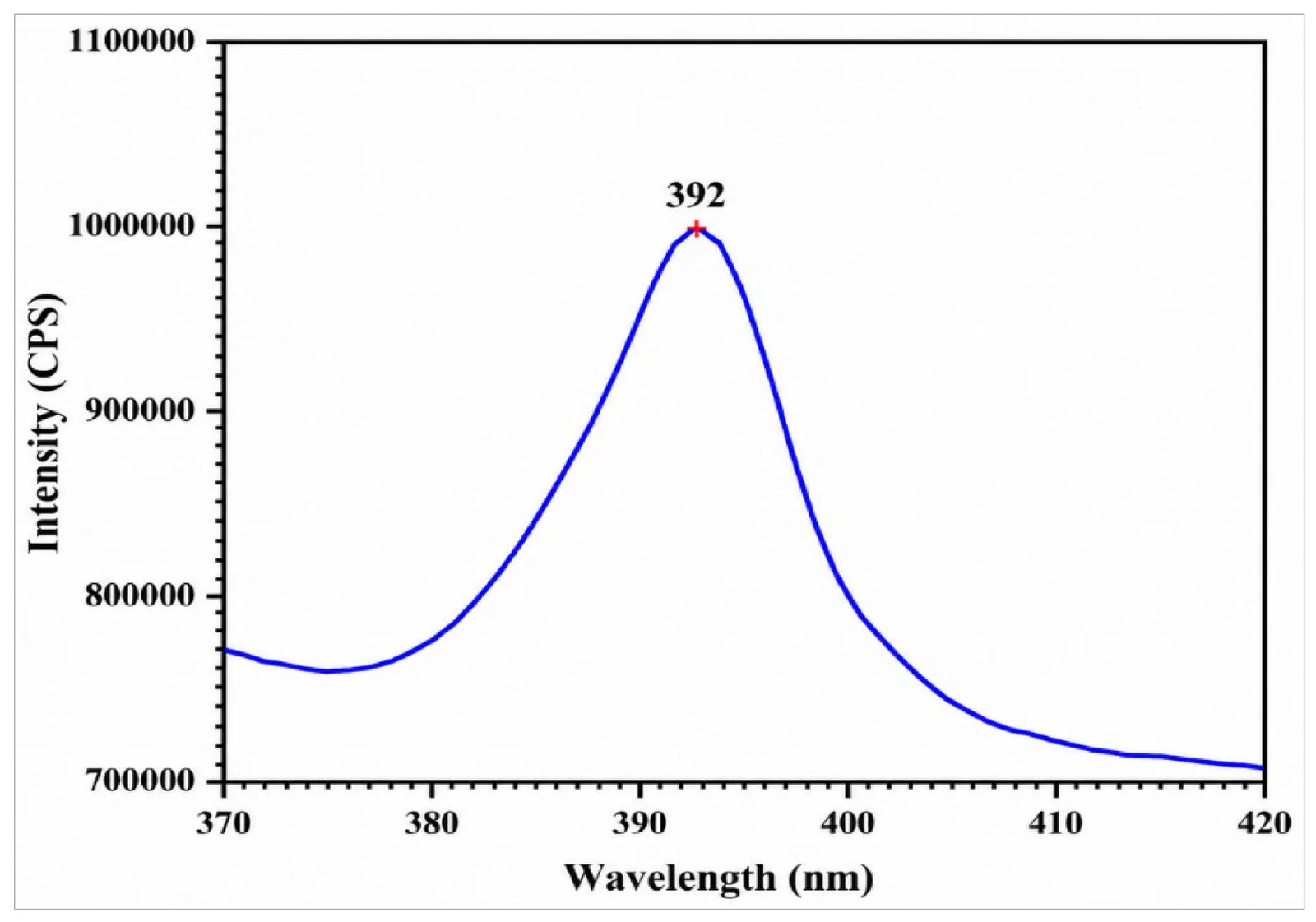
Photoluminescence spectrum of BTD.

The photoluminescence (PL) emission spectrum of the BTD crystal is shown in Figure 8. The emission spectrum was recorded using an excitation wavelength of 379 nm. A strong and broad emission band centered at 392 nm is observed, corresponding to blue emission. The blue luminescence is attributed to the electronic transitions associated with the Pb–S coordinated thiourea framework. The coordination of thiourea ligands with Pb^2+^ ions alters the molecular energy levels through ligand-to-metal charge transfer (LMCT) and intra-ligand transitions involving the sulfur lone-pair orbitals and antibonding states. The strong emission indicates efficient radiative recombination of the photo-generated charge carriers and confirms the good crystalline quality of the BTD crystal. The observed wide optical band gap of 3.27 eV, obtained from the diffuse reflectance studies, is consistent with the blue emission and suggests that the luminescence originates from near-band-edge electronic transitions. The intense blue emission demonstrates that the BTD crystal is a promising material for nonlinear optical devices and blue-light emitters [56,57,58].

### 3.8. Electronic Structure and Frontier Molecular Orbital Analysis

**Figure 9:**
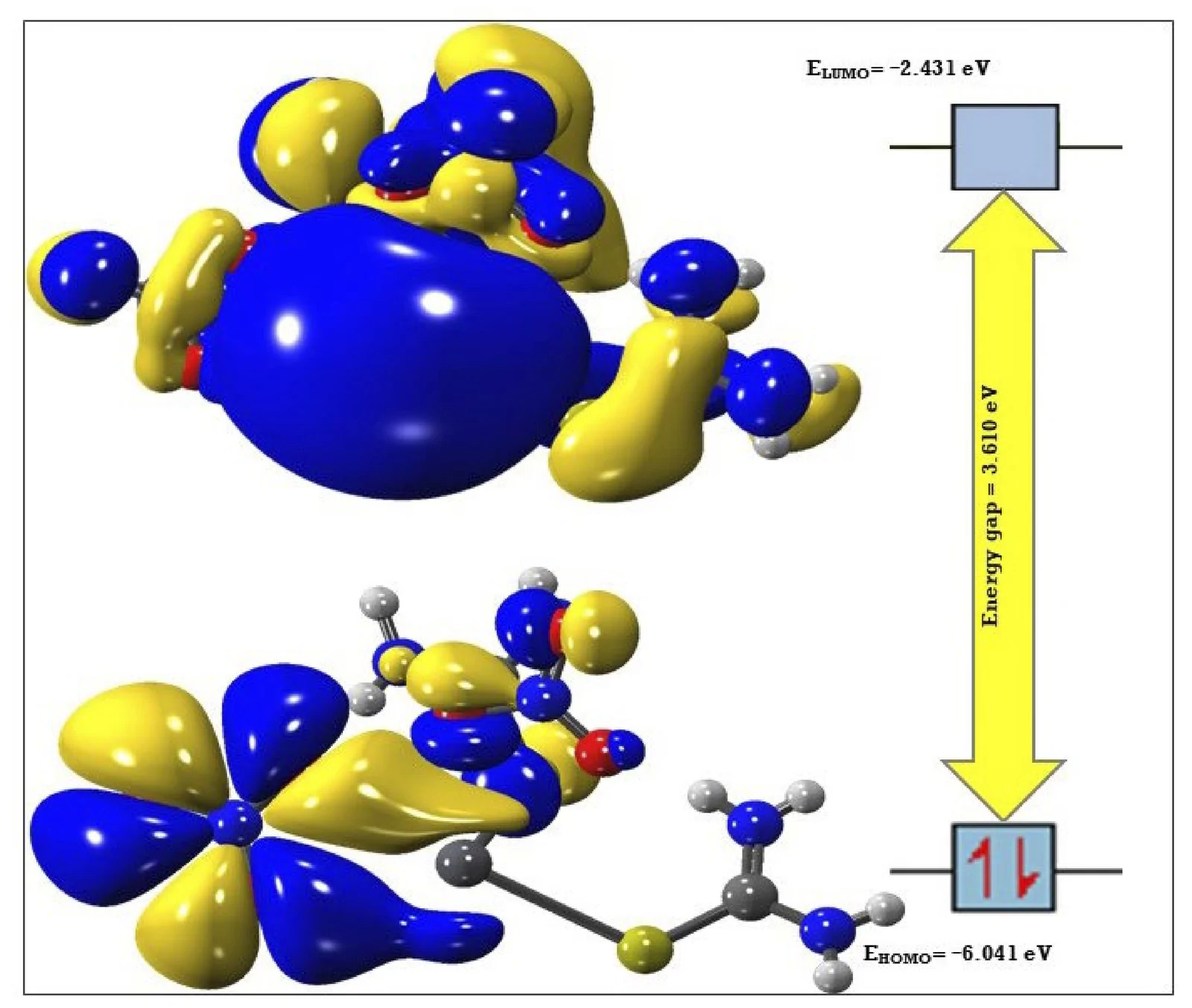
Frontier molecular orbital distributions of bis(thiourea) lead dinitrate (BTD), showing the highest occupied molecular orbital (HOMO), lowest unoccupied molecular orbital (LUMO), their corresponding orbital energies, and the calculated HOMO–LUMO energy gap.

DFT calculations were used to examine the frontier electronic structure of BTD, with the calculated HOMO and LUMO distributions shown in Figure 9. The HOMO and LUMO energies are −6.041 and −2.431 eV, respectively, giving a frontier-orbital separation of 3.610 eV. The HOMO density is concentrated mainly over the thiourea-containing regions, with substantial contribution from the sulfur-containing moieties, whereas the LUMO is distributed more extensively over the Pb–ligand framework. This spatial difference indicates that excitation is accompanied by redistribution of electron density between distinct parts of the coordinated structure.

The calculated gap is close to the experimentally determined optical band gap of 3.27 eV, although the two quantities are not directly equivalent because the former represents a molecular frontier-orbital separation whereas the latter is derived from the solid-state diffuse-reflectance spectrum. The difference of approximately 0.34 eV therefore does not indicate a direct quantitative discrepancy, but the comparable values support the wide-gap electronic character established experimentally. The spatially separated HOMO and LUMO distributions are also consistent with the experimentally observed blue photoluminescence and appreciable third-order nonlinear response, indicating that the coordinated Pb–thiourea framework participates in the electronic polarization of BTD. The orbital distributions alone, however, do not establish a specific photoluminescence or charge-transfer pathway.

### 3.9. Molecular Electrostatic Potential Analysis

**Figure 10:**
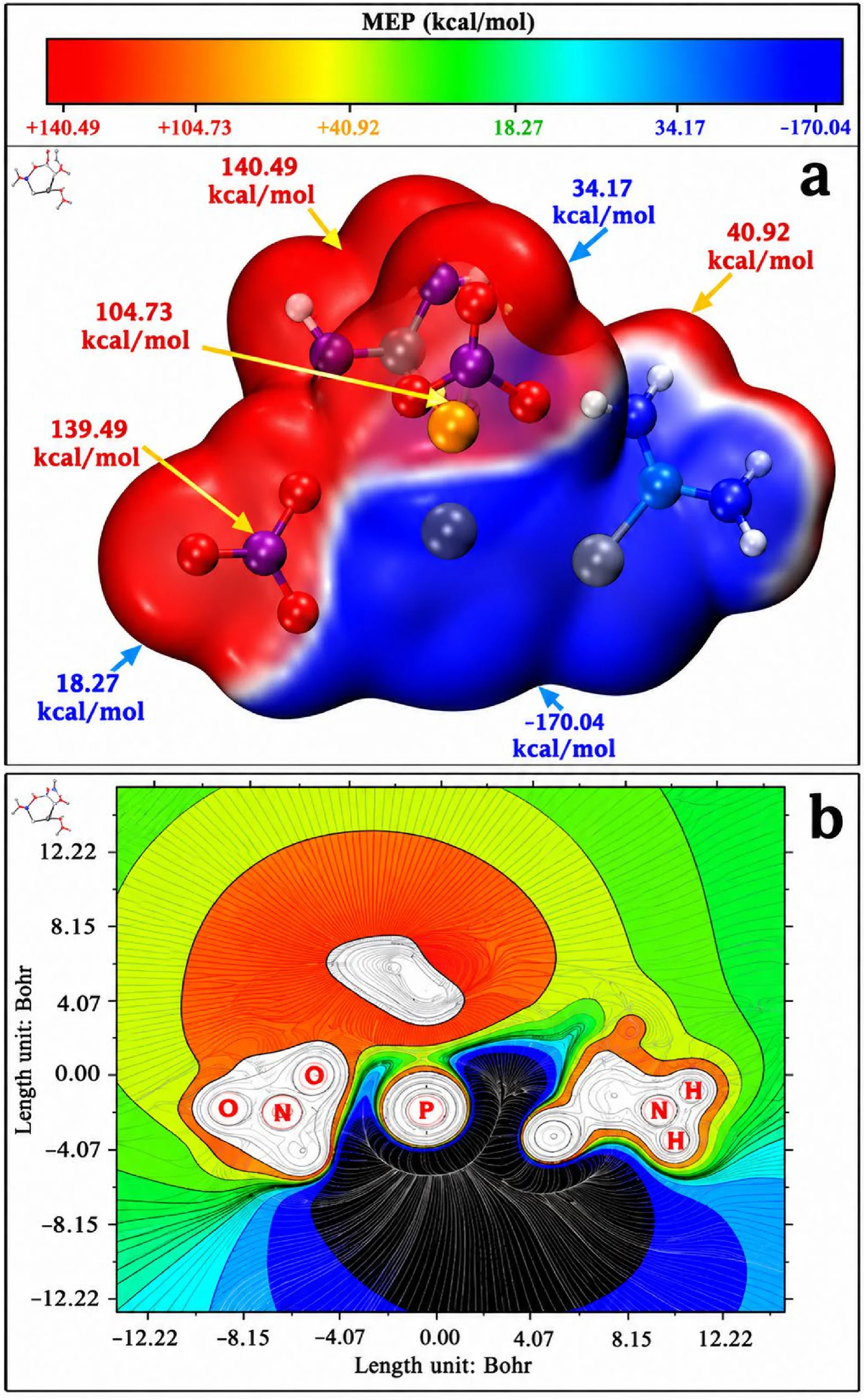
Molecular electrostatic potential (MEP) analysis of bis(thiourea) lead dinitrate (BTD): (**a**) two-dimensional electrostatic potential contour map and (**b**) MEP-mapped molecular surface showing the calculated electrostatic potential extrema.

The molecular electrostatic potential (MEP) surface of BTD, together with its contour map, is presented in Figure 10. The calculated potential spans a wide range from +140.49 to −170.04 kcal mol^−1^, indicating a pronounced variation in electrostatic character across the molecular framework. The positive and negative regions are spatially separated, showing that the charge distribution is strongly non-uniform.

The most positive regions, reaching +140.49 and +139.49 kcal mol^−1^, are concentrated around the oxygen-containing portions of the structure, while the strongly negative region reaches −170.04 kcal mol^−1^ around the central Pb-containing part of the molecular framework. Intermediate positive regions are also evident near the coordinated nitrate and thiourea groups. This distribution identifies chemically distinct sites with different electrostatic character and indicates that the Pb–ligand framework has a substantial internal electrostatic gradient.

The MEP distribution provides an electrostatic basis for the intermolecular interactions observed crystallographically. In particular, the separation of positive and negative regions is consistent with the directional N–H···O contacts identified around the nitrate groups in the crystal structure. This interpretation agrees with the Hirshfeld analysis, where O···H/H···O contacts contribute 51.6% of the intermolecular interactions. The MEP results therefore complement the crystallographic and Hirshfeld analyses by showing how the electrostatic distribution across the coordinated framework can support the observed hydrogen-bonding and packing arrangement.

### 3.10. QTAIM Analysis of the Pb Coordination Environment

**Figure 11:**
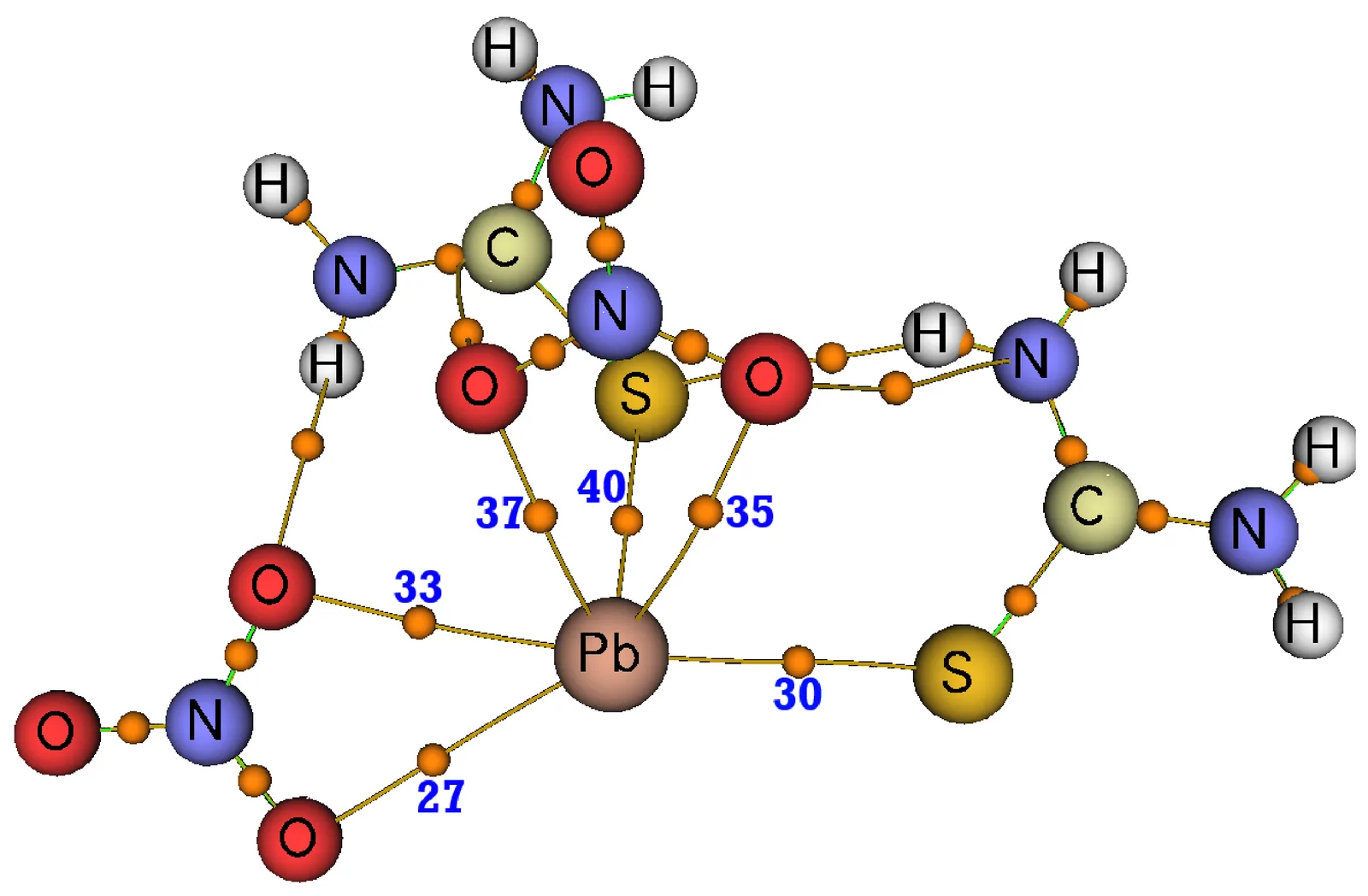
QTAIM molecular graph of bis(thiourea) lead dinitrate (BTD) showing the (3, −1) bond critical points (BCPs) associated with the Pb–O and Pb–S interactions around the Pb(II) center; the BCP numbers correspond to those listed in Table 3.

The single-crystal X-ray diffraction analysis established that the Pb(II) center in BTD is coordinated by sulfur atoms of thiourea and oxygen atoms of the nitrate groups, resulting in a distorted coordination environment. To examine the electronic characteristics of these contacts, a quantum theory of atoms in molecules (QTAIM) analysis was performed. The bond critical points (BCPs) identified around the Pb center are shown in Figure 11, and the corresponding topological parameters are listed in Table 3. Six (3, −1) critical points were identified, comprising four Pb–O and two Pb–S contacts. Their presence confirms that the crystallographically observed coordination contacts are also associated with well-defined features in the calculated electron density.

The electron density, ρ(r), at the BCPs varies among the individual coordination contacts, with values of 0.02424–0.03830 for Pb–O and 0.03364–0.03640 for Pb–S interactions. The highest value occurs at BCP 37 for Pb–O, followed by BCP 40 for Pb–S, indicating greater electron-density concentration at these two contacts relative to the other Pb–O interactions. The variation in ρ(r) is consistent with the non-equivalent Pb–O and Pb–S contacts evident from the crystal structure and supports the electronically differentiated nature of the Pb coordination sphere. Thus, the distortion observed crystallographically is accompanied by differences in the local electron-density distribution around the metal center.

Further information is obtained from the Laplacian of the electron density and the local energy-density parameters. All six BCPs have positive ∇^2^ρ values, ranging from 0.06169 to 0.13080, while G(r) is positive and V(r) is negative for every Pb–O and Pb–S contact. The magnitude of the negative potential-energy density is largest for BCP 37, consistent with its relatively high electron density. The combination of positive ∇^2^ρ, positive G(r), and negative V(r) indicates that the Pb–O and Pb–S contacts do not exhibit the electron-density concentration expected for strong shared-shell interactions. At the same time, the negative V(r) values show that the electron-density distribution at each critical point contributes to local stabilization of the coordination environment. The |V(r)|/G(r) values (Table 3) lie between approximately 1.07 and 1.22, supporting the view that these contacts have a predominantly closed-shell character within the present QTAIM description rather than behaving as conventional covalent bonds. In addition, the total energy density H(r) = G(r) + V(r) is slightly negative at all six BCPs (−0.0011 to −0.0036 a.u.), while 1 < |V(r)|/G(r) < 2. These values place the Pb–O and Pb–S contacts in the intermediate regime between pure closed-shell and shared-shell interactions, i.e., predominantly closed-shell (ionic) interactions with a small covalent contribution.

The QTAIM results consequently provide an electronic description of the distorted Pb(II) coordination environment identified by SCXRD. The unequal ρ(r), ∇^2^ρ, G(r), and V(r) values show that the four Pb–O and two Pb–S contacts are not electronically equivalent, with BCP 37 representing the most pronounced Pb–O interaction among those examined. This distinction is important because the crystal structure is not determined solely by the geometry of the coordination sphere; the individual Pb–O and Pb–S contacts make different electronic contributions to the local environment of Pb(II). In conjunction with the MEP analysis, these results provide a more detailed basis for relating the coordination structure to the electronic characteristics of BTD. The analysis also complements the Hirshfeld result showing a dominant contribution from O···H/H···O contacts (51.6%), although QTAIM here specifically characterizes the Pb coordination contacts rather than the intermolecular hydrogen-bond network. The nature of the broader attractive and repulsive interactions can therefore be examined further using NCI–RDG analysis.

### 3.11. Non-Covalent Interaction Analysis

**Figure 12:**
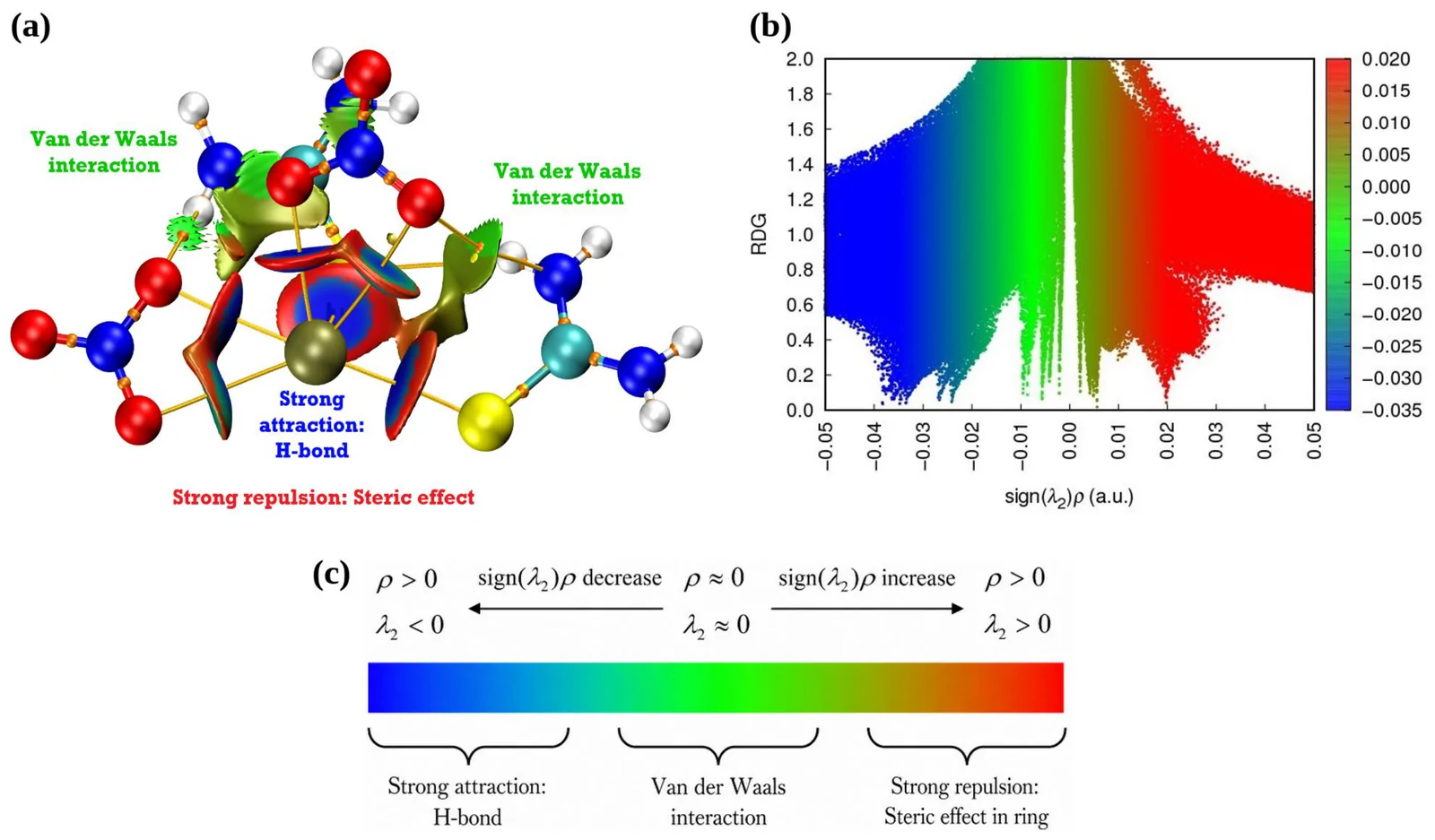
Non-covalent interaction (NCI) analysis of BTD: (**a**) reduced density gradient (RDG) isosurface coloured by sign(λ_2_)ρ, showing the hydrogen-bonding, van der Waals and steric regions around the Pb(II) coordination sphere; (**b**) RDG versus sign(λ_2_)ρ (a.u.) scatter plot; (**c**) color scale used to classify the interactions (blue, strong attraction such as hydrogen bonding; green, van der Waals interactions; red, strong steric repulsion).

To further characterize the intermolecular interactions identified from the crystal structure and Hirshfeld surface analysis, a reduced density gradient (RDG)-based non-covalent interaction (NCI) analysis was performed. The NCI isosurface and corresponding RDG versus sign(λ_2_)ρ plot are shown in Figure 12. The color distribution separates the interaction regions according to the sign and magnitude of λ_2_ρ, with negative values corresponding to attractive interactions, values close to zero representing weak van der Waals contacts, and positive values indicating repulsive regions. The calculated map therefore provides a real-space view of the different interactions operating within the BTD molecular assembly.

Distinct blue regions are observed between the interacting molecular fragments and are assigned to strong attractive interactions, with the figure specifically identifying a pronounced region associated with hydrogen bonding. These regions correspond to the negative sign(λ_2_)ρ domain in the RDG plot. The presence of these attractive regions agrees with the crystal structure described in Section 3.3, where N–H···O contacts between thiourea amino groups and nitrate oxygen atoms were identified as important contributors to the supramolecular arrangement. The NCI result consequently provides electronic evidence that the close contacts observed crystallographically are associated with attractive interaction regions rather than arising solely from geometrical packing.

The NCI map also contains green regions centered near sign(λ_2_)ρ ≈ 0, corresponding to van der Waals interactions, as indicated in Figure 12. These weaker contacts occur alongside the stronger attractive regions and contribute to the packing environment without being assigned the same interaction character as the hydrogen bonds. In contrast, the red regions at positive sign(λ_2_)ρ correspond to repulsive interactions, including the steric regions identified within the molecular framework. The coexistence of attractive, weakly dispersive and repulsive regions reflects the balance of interactions required to accommodate the molecular components within the crystal structure.

The NCI analysis complements the Hirshfeld surface results in a useful way. Hirshfeld analysis showed that O···H/H···O contacts contribute 51.6% of the intermolecular contacts in BTD, identifying hydrogen-containing oxygen contacts as the major contribution to the crystal packing. NCI analysis does not provide the same percentage-based contact distribution; instead, it reveals the spatial character of the interactions and distinguishes the attractive hydrogen-bonding regions from weaker van der Waals and repulsive regions. The agreement between these two analyses strengthens the interpretation that hydrogen bonding is an important feature of the BTD supramolecular framework, while the coexistence of weaker and repulsive regions helps define the packing arrangement established by the crystal structure.

### 3.12. Real-Space Electron-Density Analysis

**Figure 13:**
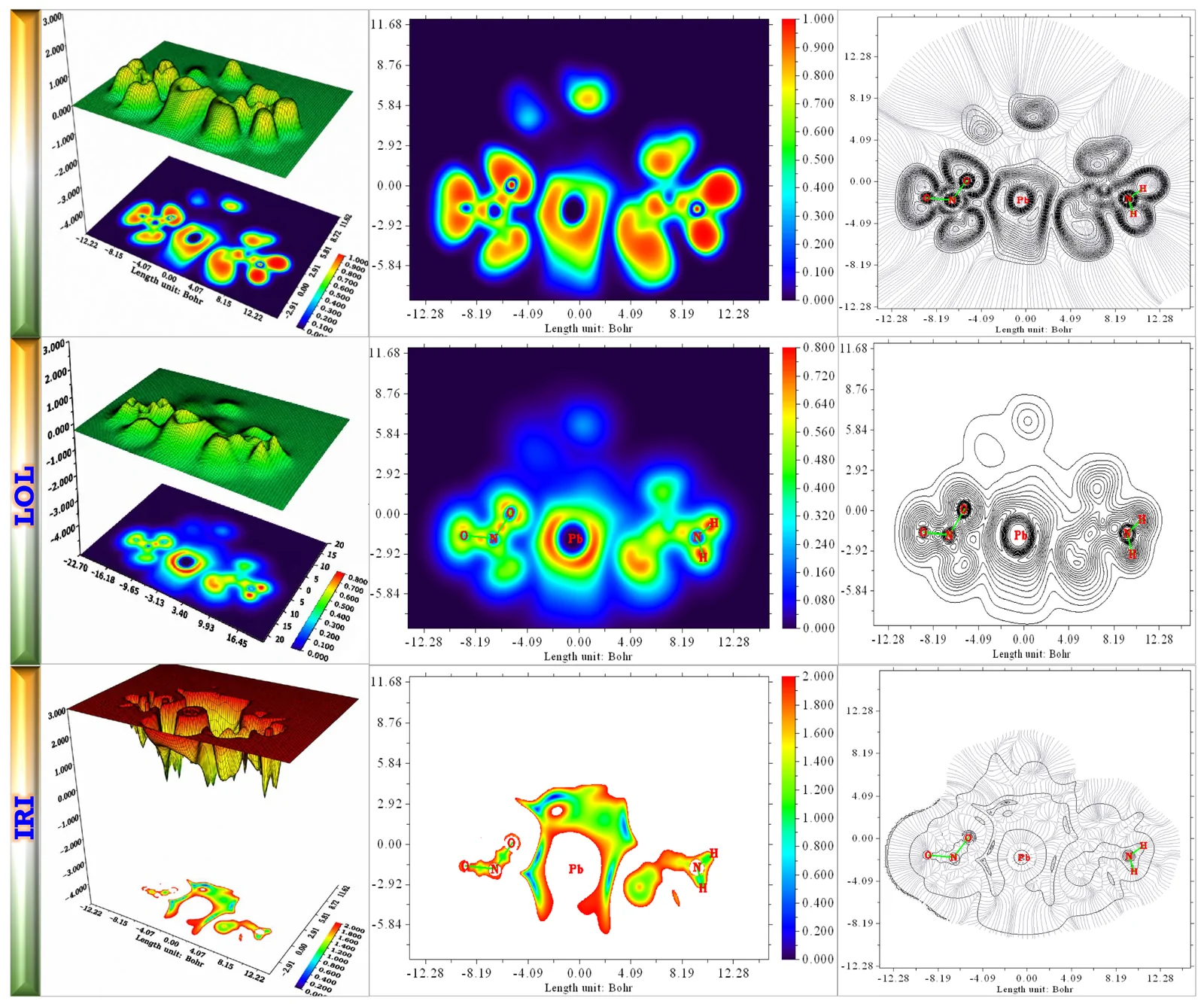
Real-space electron-density descriptors for BTD mapped onto the electron-density surface, showing the electron localization function (ELF), localized orbital locator (LOL), and interaction region indicator (IRI) distributions.

The real-space distribution of electron density in BTD was further examined using the electron localization function (ELF), localized orbital locator (LOL) and interaction region indicator (IRI) descriptors, as presented in Figure 13. The three descriptors provide complementary views of the electron distribution around the Pb-centered coordination framework. The ELF maps show pronounced localized regions around the constituent atoms, with continuous features extending towards the Pb center from the surrounding donor atoms. These features are consistent with the coordination environment identified by SCXRD and the presence of Pb–O and Pb–S critical points established by QTAIM.

The LOL distribution gives a similar spatial representation of the localization within the ligand framework, while also showing distinct regions around the Pb center and the coordinating atoms. The localization patterns are not uniform across the structure, reflecting the different electronic environments of the O-, N- and S-containing groups. In particular, the regions extending between Pb and the coordinating atoms support the presence of electronic interaction within the coordination sphere rather than an isolated Pb center. The IRI distribution further outlines the electron-density regions surrounding Pb and the neighboring ligand atoms, providing complementary evidence for the redistribution of electron density across the metal–ligand framework.

These real-space maps are consistent with the QTAIM results, in which Pb–O and Pb–S contacts were associated with identifiable (3, −1) critical points and non-equivalent electron-density characteristics. The agreement between the spatial localization maps and the topological analysis supports the crystallographically established coordination arrangement without requiring the Pb–O and Pb–S contacts to be assigned an identical bonding character. The non-uniform electron localization around the coordination sphere also provides a basis for considering the Pb-containing framework in relation to the electronic polarization responsible for the optical properties of BTD. Together with the HOMO–LUMO and MEP results, these observations complete the electronic-structure description before consideration of the intermolecular contact contributions obtained from Hirshfeld surface analysis.

### 3.13. Hirshfeld Surface Analysis

**Figure 14:**
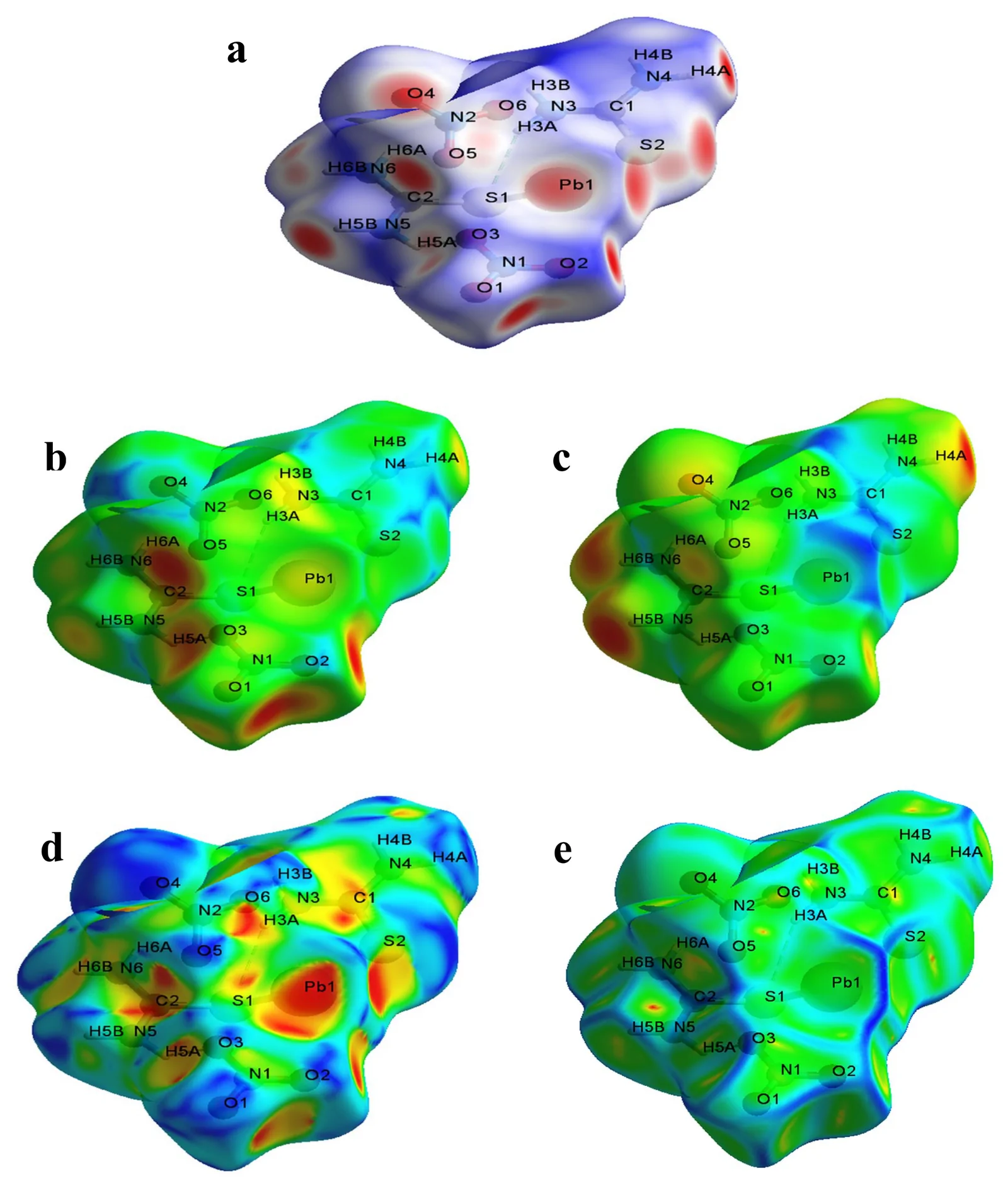
Hirshfeld surfaces (**a**) The *d*_norm_ surface overlaid on a representation of the interactions determining the crystal packing. Surfaces for (**b**) *d*_i_, (**c**) *d*_e_, (**d**) shape index and (**e**) curvedness.

**Figure 15:**
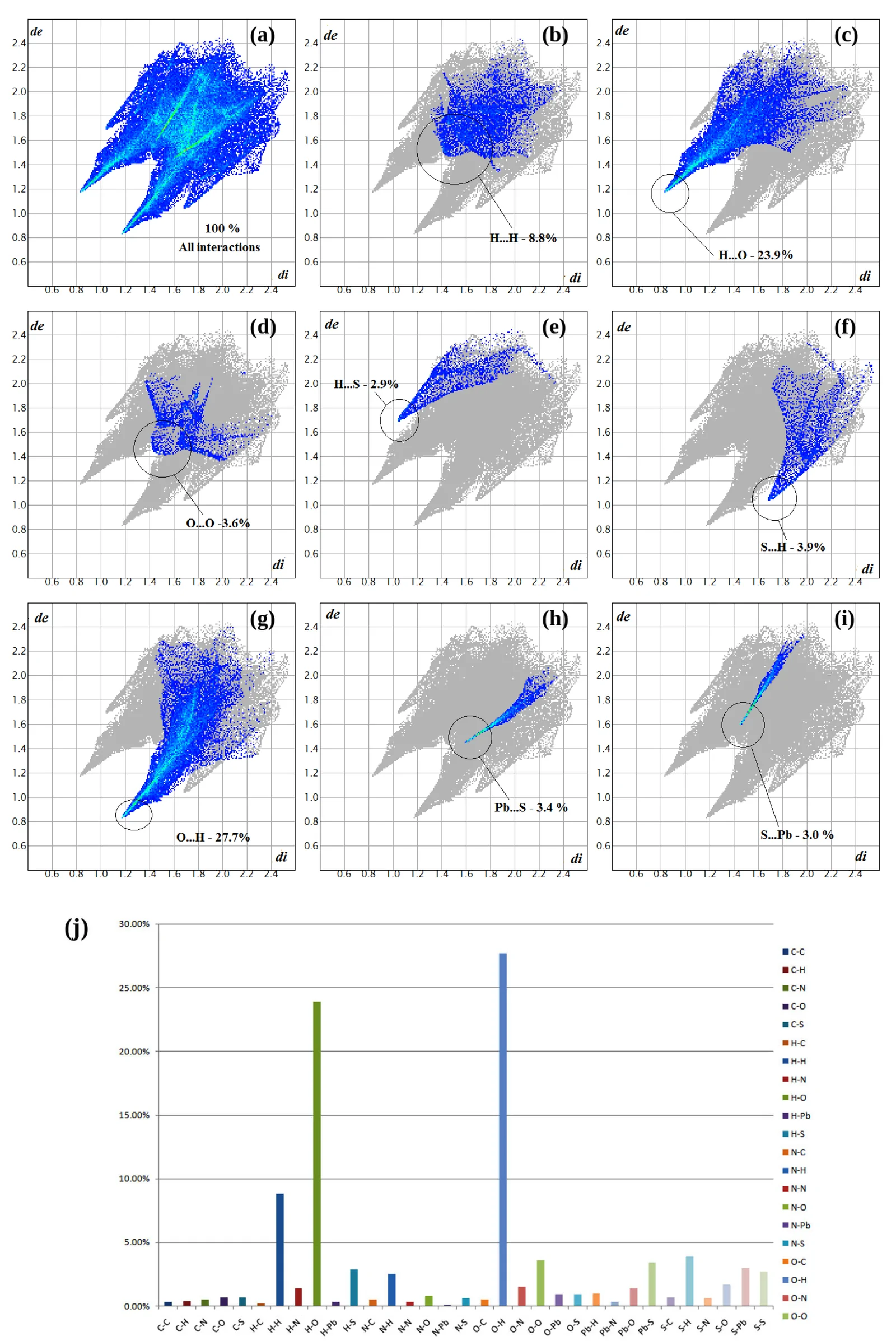
Two-dimensional fingerprint plots of BTD: (**a**) all intermolecular contacts (100%) and the decomposed contributions of (**b**) H···H (8.8%), (**c**) H···O (23.9%), (**d**) O···O (3.6%), (**e**) H···S (2.9%), (**f**) S···H (3.9%), (**g**) O···H (27.7%), (**h**) Pb···S (3.4%) and (**i**) S···Pb (3.0%); (**j**) bar chart of the percentage contributions of the individual intermolecular contacts. d_i_ and d_e_ (Å) are the distances from the Hirshfeld surface to the nearest nucleus inside and outside the surface, respectively.

The intermolecular interactions responsible for the crystal packing of BTD were investigated using Hirshfeld surface analysis [59,60]. The Hirshfeld surfaces mapped over *d*_norm_, shape index, *d*_e_, *d*_i_, and curvedness are presented in Figure 14, while the corresponding two-dimensional (2D) fingerprint plots [61] are shown in Figure 15.

The *d*_norm_ surface displays several intense red regions, indicating short intermolecular contacts associated with strong N–H···O and N–H···S hydrogen bonds. White regions represent contacts close to the van der Waals radii, whereas the blue regions correspond to longer intermolecular contacts. The shape index and curvedness surfaces reveal no significant complementary red–blue triangular patterns, indicating the absence of appreciable π···π stacking interactions. Therefore, the crystal packing is predominantly governed by hydrogen bonding.

The quantitative contributions of individual intermolecular contacts obtained from the decomposed fingerprint plots are summarized in Figure 15. The O···H/H···O interactions constitute the largest contribution (51.6%), comprising O···H (27.7%) and H···O (23.9%), confirming that hydrogen bonding is the primary stabilizing force in the crystal structure. The H···H contacts contribute 8.8%, representing weak van der Waals interactions. Smaller contributions arise from O···O (3.6%), H···S (2.9%), S···H (3.9%), Pb···S (3.4%), and S···Pb (3.0%), indicating additional weak contacts that further reinforce the three-dimensional supramolecular framework. The percentage contributions of all intermolecular contacts are compared in the bar chart in Figure 15j.

The predominance of O···H/H···O interactions demonstrates the formation of an extensive hydrogen-bonding network, which enhances crystal packing stability and structural rigidity. This robust intermolecular network is consistent with the observed thermal stability and contributes to efficient electronic polarization, supporting the excellent optical transparency and appreciable third-order nonlinear optical response of the BTD crystal.

### 3.14. Third-Order Nonlinear Optical (Z-Scan) Studies

**Figure 16:**
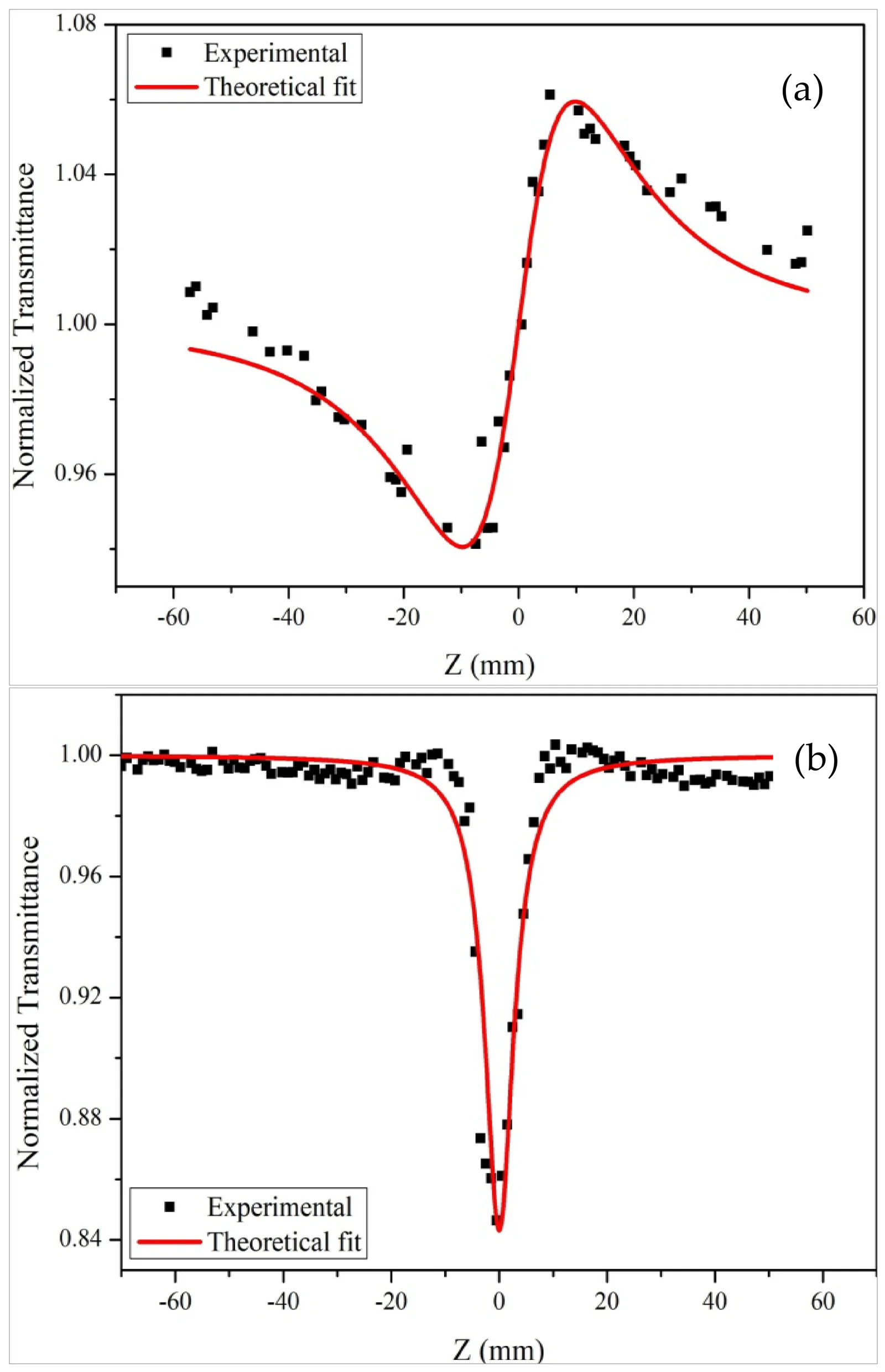
Zscan transmittance curves of BTD: (**a**) closed aperture and (**b**) open aperture.

The third-order nonlinear optical (NLO) properties of the BTD crystal were investigated using the Z-scan technique with a pulsed Nd:YAG laser at 532 nm. The Z-scan method is a simple and sensitive technique used to determine the nonlinear refractive index (n_2_) and nonlinear absorption coefficient (*β*) of a material by translating the sample through the focal region of a Gaussian laser beam. The closed-aperture (CA) configuration measures nonlinear refraction, whereas the open-aperture (OA) configuration determines nonlinear absorption. The results of the Z-scan curves with and without aperture (closed and open aperture) are shown in Figure 16.

The nonlinear refractive index and nonlinear absorption coefficient were calculated using the standard Z-scan relations:

(1) $$\Delta T_{p-v}=0.406(1-S{)}^{0.25}|\Delta \Phi_{0}|$$

(2) $$n_{2}=\frac{\Delta \Phi_{0}}{kI_{0}L_{eff}}$$

(3) $$\beta =\frac{2\sqrt{2}\Delta T}{I_{0}L_{eff}}$$

where $\Delta T_{p-v}$ is the peak-to-valley transmittance difference, S is the aperture linear transmittance, $\Delta \Phi_{0}$ is the on-axis phase shift, k=2π/λ is the wave vector, $I_{0}$ is the laser intensity at focus, and $L_{eff}=[1-exp(-\alpha L)]/\alpha$ is the effective sample length.

The third-order nonlinear susceptibility was obtained from

(4) $$|\chi^{\left(3\right)}|={\left[{\left(Re\;\chi^{\left(3\right)}\right)}^{2}|{\left(Im\;\chi^{\left(3\right)}\right)}^{2}\right]}^{1/2}$$

The open- and closed-aperture Z-scan curves are shown in Figure 16, and the calculated third-order NLO parameters are listed in Table 4. The closed-aperture trace exhibits a characteristic peak–valley profile, indicating a positive nonlinear refractive index (n_2_ = 2.7185 × 10^−19^ m^2^ W^−1^) and confirming the self-focusing behavior of the crystal. The open-aperture curve shows a transmission minimum at the focal position, revealing reverse saturable absorption (RSA) with a nonlinear absorption [62,63] coefficient of *β* = 3.0988 × 10^−12^ m W^−1^.

The calculated values of Reχ^(3)^ = 0.8924 × 10^−13^ esu, Imχ^(3)^ = 0.4309 × 10^−13^ esu, and χ^(3)^ = 0.9909 × 10^−13^ esu demonstrate a significant third-order nonlinear optical response. The enhanced nonlinearity is attributed to the Pb–S coordination, efficient ligand-to-metal charge transfer, and the extensive intra- and intermolecular hydrogen-bonding network, which increase the electronic polarizability of the crystal.

The third-order nonlinear susceptibility of the BTD crystal (0.9909 × 10^−13^ esu) is comparable to or slightly higher than that of several reported thiourea-based semiorganic crystals [12,15,28,29] investigated under similar experimental conditions (Table 5). The enhanced nonlinear response can be attributed to the high electronic polarizability of the Pb^2+^ ion, strong Pb–S coordination, efficient ligand-to-metal charge transfer, and the extensive hydrogen-bonding network within the crystal structure. In addition to its appreciable χ^(3)^ value, BTD exhibits reverse saturable absorption, self-focusing behavior, and a wide optical band gap (3.27 eV), making it a promising material for optical limiting, all-optical switching, and photonic device applications.

Compared with previously reported thiourea-based Pb(II) coordination crystals, BTD exhibits a competitive third-order nonlinear susceptibility together with pronounced reverse saturable absorption and positive nonlinear refraction. The enhanced nonlinear response can be attributed to the synergistic contribution of the highly polarizable Pb(II) center, efficient Pb–S coordination, ligand-to-metal charge transfer, and the extensive hydrogen-bonding network revealed by the Hirshfeld surface analysis. These structural features increase the electronic polarizability and facilitate nonlinear optical interactions, resulting in improved optical limiting performance. The combined experimental and theoretical results demonstrate that BTD possesses a favorable structure–property relationship for nonlinear photonic applications.

The third-order response of metal–thiourea crystals is governed mainly by three structural factors. (i) Metal–ligand coordination: thiourea binds through its soft sulfur donor, and the electron density of the S=C–N π-system is delocalized towards the metal through the M–S bond; the more polarizable and covalent this bond is, the larger the induced dipole under intense optical fields. In BTD, Pb(II) is bonded to two thiourea S atoms (Pb–S = 2.85–3.05 Å) and to nitrate O atoms, and the QTAIM analysis (Table 3) shows negative H(r) and |V(r)|/G(r) > 1 at all six BCPs, indicating a partial covalent contribution that favors charge redistribution within the coordination sphere. (ii) Hydrogen-bond network: in the chloride-based crystals BTCC and BTZC, the thiourea N–H groups are linked through N–H···Cl contacts, whereas in BTD they form N–H···O hydrogen bonds with the planar, π-conjugated nitrate anions (O···H/H···O = 51.6% of the Hirshfeld surface), which couples the polarizable NO_3_^−^ groups to the thiourea π-system throughout the lattice. (iii) Metal lone-pair effect: the d^10^ ions Zn^2+^ and Cd^2+^ in BTZC, BTZA, BTCC and ZTS have no stereochemically active lone pair and adopt tetrahedral geometries, whereas the 6s^2^ lone pair of Pb^2+^ distorts the coordination polyhedron (S–Pb–S = 80.46°), creating an asymmetric charge distribution and a highly polarizable metal center, as also observed in other Pb-based NLO crystals [30,31]; the heavy-atom character of Pb^2+^ additionally favors excited-state absorption, consistent with the reverse saturable absorption observed here. Because χ^(3)^ is not restricted by crystal symmetry, the centrosymmetric P2_1_/c structure does not limit these contributions. Together, these factors account for the larger χ^(3)^ of BTD (0.99 × 10^−13^ esu) compared with BTCC (0.65), BTZC (0.74), BTZA (0.82) and ZTS (0.91 × 10^−13^ esu).

### 3.15. Optical Limiting Studies

**Figure 17:**
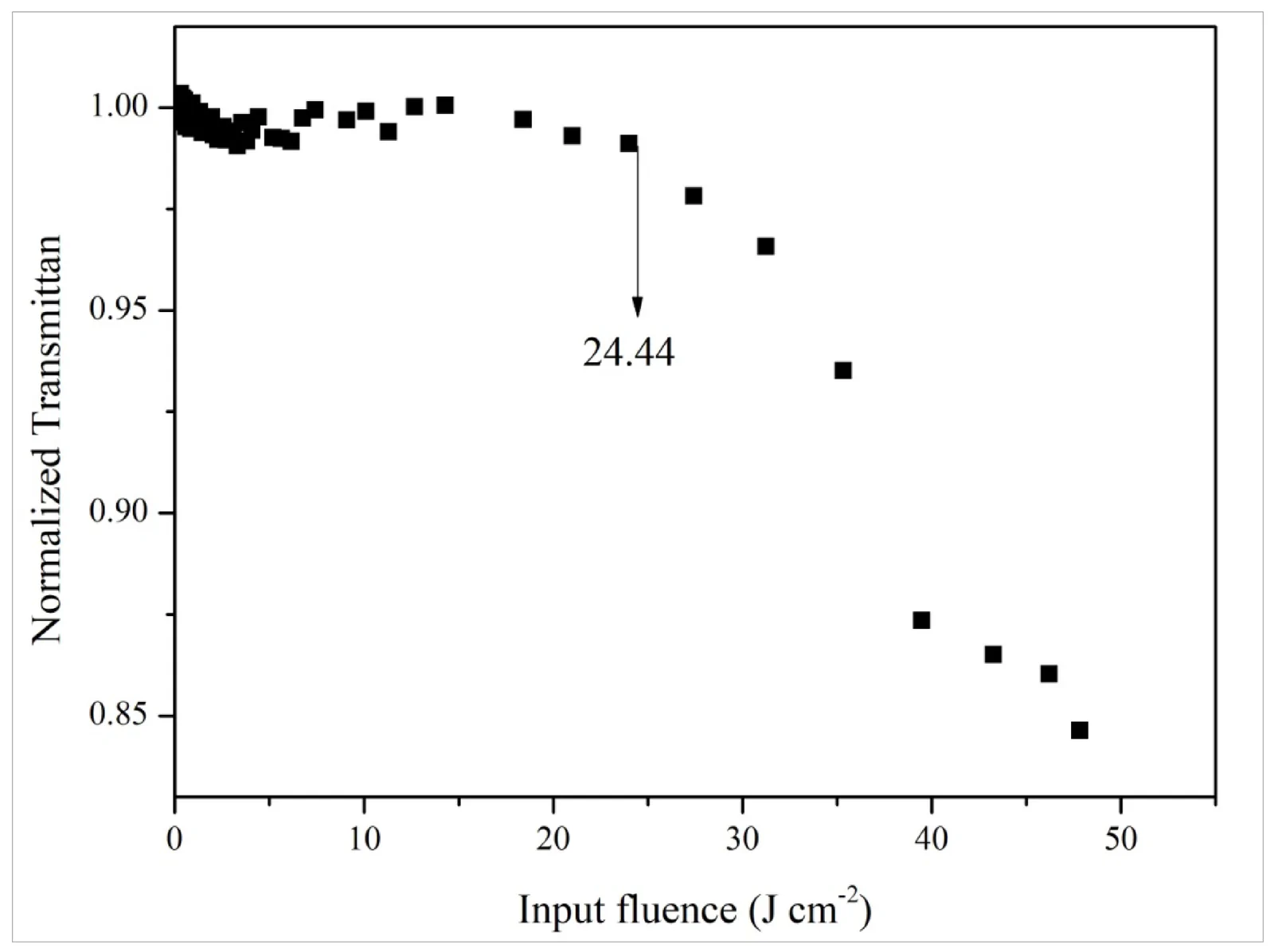
Optical limiting curve of BTD.

The optical limiting behavior of the BTD crystal was evaluated from the open-aperture Z-scan measurements, and the corresponding optical limiting curve is shown in Figure 17. Optical limiting is an important nonlinear optical property in which a material exhibits high transmission at low incident laser fluence but suppresses transmission as the incident fluence increases, thereby protecting optical sensors and human eyes from high-intensity laser radiation.

The BTD crystal exhibits nearly constant transmittance at low input fluence, indicating negligible nonlinear absorption. With increasing laser fluence, the normalized transmittance decreases progressively due to the onset of reverse saturable absorption (RSA). The optical limiting threshold is observed at 24.44 J cm^−2^, beyond which the transmission decreases rapidly, demonstrating efficient attenuation of the incident laser beam.

The excellent optical limiting performance of the BTD crystal is closely associated with its appreciable third-order nonlinear susceptibility (χ^(3)^ = 0.9909 × 10^−13^ esu) and positive nonlinear refractive index obtained from the Z-scan analysis. The combination of strong nonlinear absorption, wide optical transparency, and a relatively low limiting threshold demonstrates that the BTD crystal is a promising material for laser protection, optical limiting, optical sensor protection, and all-optical photonic switching applications.

### 3.16. Band Structure and Density of States

**Figure 18:**
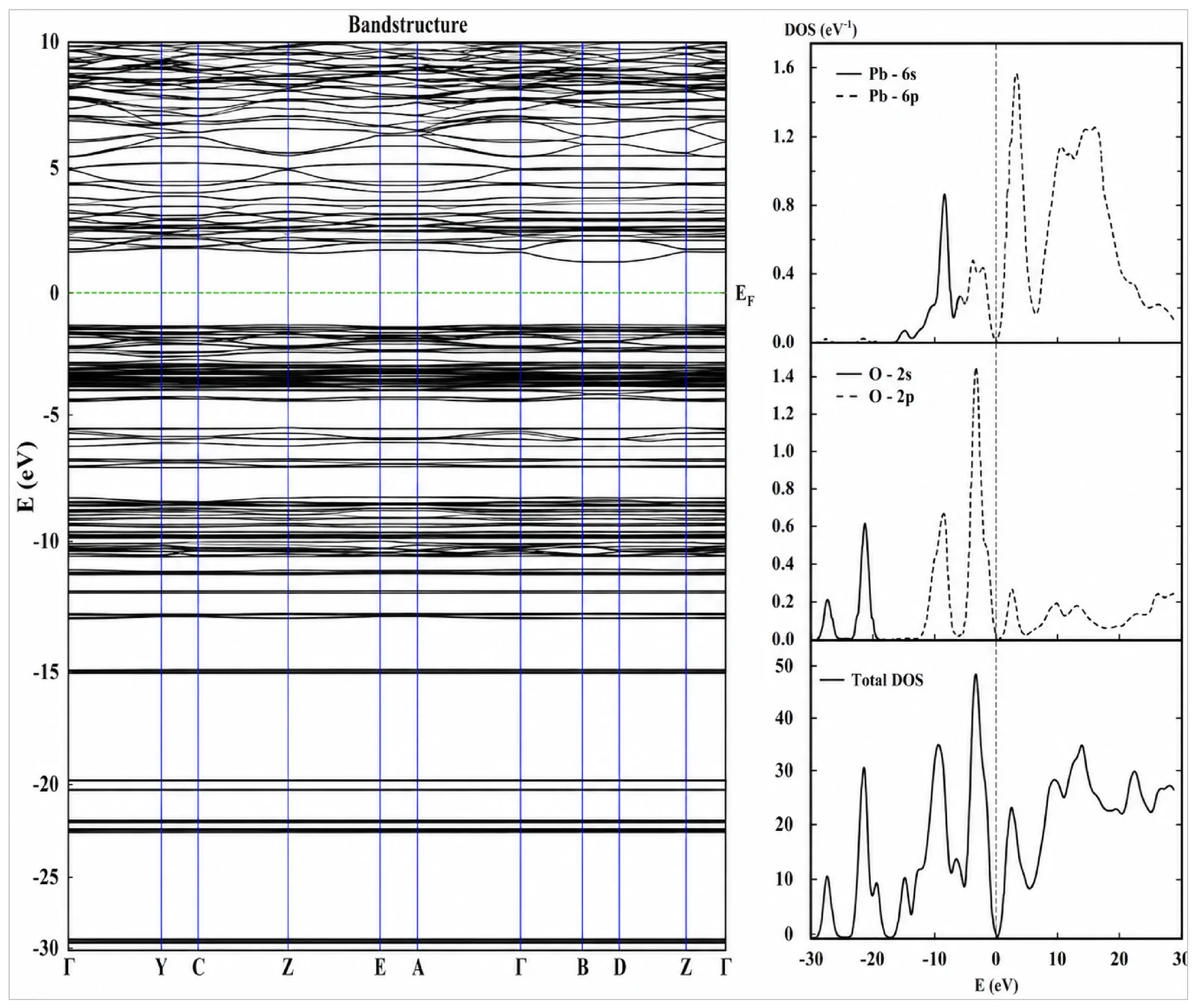
Calculated band structure of BTD and total and partial density of states of BTD.

The electronic band structure and density of states (DOS) were calculated using density functional theory (DFT) to understand the electronic structure and optical behavior of the BTD crystal. The calculated band structure along the high-symmetry directions of the Brillouin zone is presented in Figure 18. The valence electron configurations considered in the calculations were Pb (6s^2^6p^2^), S (3s^2^3p^4^), O (2s^2^2p^4^), N (2s^2^2p^3^), C (2s^2^2p^2^) and H (1s^1^).

The band structure reveals that the valence band maximum (VBM) and conduction band minimum (CBM) occur at the same k-point, confirming the direct band-gap nature of the crystal. The calculated band gap is 2.95 eV, about 0.32 eV smaller than the experimentally determined optical band gap of 3.27 eV obtained from diffuse reflectance measurements. Such underestimation is well known for conventional (semi-)local exchange–correlation functionals: the Kohn–Sham eigenvalue gap does not include the derivative discontinuity of the exact exchange–correlation potential [64,65], and the delocalization (self-interaction) error of approximate functionals further narrows the gap [66]. Screened hybrid functionals such as HSE06 [67] or many-body GW corrections [68] generally bring calculated gaps closer to experiment. In addition, the experimental value is an optical gap derived from Kubelka–Munk/Tauc analysis at room temperature, whereas the calculation refers to a static lattice, so a modest difference is expected. The direct character of the gap and the orbital composition of the band edges are not affected by this systematic offset.

The total and partial density of states (DOS) are shown in Figure 18. The upper valence band is dominated by O-2p states with a minor contribution from Pb-6s orbitals, whereas the lower valence band mainly consists of O-2s states. The conduction band is primarily composed of Pb-6p states with contributions from O-2p orbitals. These results indicate that the fundamental optical transition predominantly occurs from the O-2p-derived valence band to the Pb-6p-derived conduction band, reflecting charge transfer between the oxygen and lead atoms.

The strong O-2p and Pb-6p character of the band-edge states enhances electronic polarization and facilitates optical excitation, which is consistent with the experimentally observed wide optical transparency, strong photoluminescence, and appreciable third-order nonlinear optical response. Thus, the DFT calculations provide theoretical support for the excellent optical and photonic characteristics of the BTD crystal.

## 4. Conclusions

Single crystals of bisthiourea lead dinitrate (BTD) were successfully grown by the slow evaporation solution growth technique at room temperature. Single-crystal X-ray diffraction analysis confirmed the crystal structure, while powder X-ray diffraction verified the phase purity and good crystallinity of the grown crystal. FT-IR spectroscopy confirmed the coordination of thiourea with Pb^2+^ through sulfur atoms and verified the presence of the characteristic functional groups. SEM analysis revealed a homogeneous surface morphology with well-developed crystal growth. Thermal analysis (TG–DTA) demonstrated that the crystal is thermally stable up to 188 °C, making it suitable for device operation below its decomposition temperature. Diffuse reflectance studies showed excellent optical transparency in the visible region with a wide direct optical band gap of 3.27 eV. Photoluminescence measurements exhibited an intense blue emission centered at 392 nm, indicating efficient radiative recombination and good optical quality. Density functional theory (DFT) calculations confirmed the direct band-gap nature of BTD with a calculated band gap of 2.95 eV. Hirshfeld surface analysis established that the crystal packing is predominantly stabilized by O···H/H···O hydrogen-bonding interactions, contributing to the structural stability of the crystal. Z-scan measurements demonstrated a positive nonlinear refractive index, reverse saturable absorption, and an appreciable third-order nonlinear susceptibility of 0.9909 × 10^−13^ esu, together with an optical limiting threshold of 24.44 J cm^−2^. The combined results demonstrate that the BTD crystal possesses excellent structural, thermal, optical, and nonlinear optical characteristics, making it a promising candidate for optical limiting, all-optical switching, nonlinear photonic, and optoelectronic device applications.

## Figures and Tables

**Table 1:** Assignment of the characteristic FT-IR vibrational bands of bis(thiourea) lead dinitrate (BTD).

Wavenumber (cm^−1^)	Assignment
3395, 3298	ν(N–H) asymmetric/symmetric stretching
3194	ν(N–H) stretching
1621	δ(NH_2_) bending
1525	ν(C–N) stretching
1378	ν(NO_3_^−^) symmetric stretching
1098	ν(C=S) stretching
1041	ν(C–N) stretching
819	N–C–S bending
696	C=S deformation
655	N–C–S deformation

**Table 2:** Crystallographic data and structure refinement parameters for bis(thiourea) lead dinitrate (BTD).

Parameter	Value
Empirical formula	C_2_H_8_N_6_O_6_PbS_2_
Formula weight	483.45
Temperature (K)	296(2)
Wavelength (Å)	0.71073
Crystal system	Monoclinic
Space group	*P*2_1_/*c*
**Unit cell dimensions**	
*a* (Å)	7.2167(3)
*b* (Å)	17.9649(8)
*c* (Å)	9.4902(5)
*α* (°)	90
*β* (°)	108.552(2)
*γ* (°)	90
Volume (Å^3^)	1166.44(9)
*Z*	4
Calculated density (Mg m^−3^)	2.753
Absorption coefficient, μ (mm^−1^)	14.85
*F*(000)	896
Crystal size (mm^3^)	0.400 × 0.300 × 0.150
θ range for data collection (°)	2.977–29.995
Index ranges	−10 ≤ *h* ≤ 9, −25 ≤ *k* ≤ 25, −13 ≤ *l* ≤ 13
Reflections collected	18,647
Independent reflections	3394 [*R*int = 0.0736]
Completeness to θ = 25.242° (%)	99.9
Refinement method	Full-matrix least-squares on *F*^2^
Data/restraints/parameters	3394/0/187
Goodness-of-fit on *F*^2^	1.156
Final *R* indices [*I* > 2σ(*I*)]	*R*1 = 0.0426, w*R*2 = 0.0636
*R* indices (all data)	*R*1 = 0.0693, w*R*2 = 0.0697
Extinction coefficient	0.0055(2)
Largest difference peak and hole (e Å^−3^)	1.719 and −1.145
**CCDC number**	**1** **,** **554** **,** **847**

**Table 3:** Topological parameters (a.u.) at the (3, −1) bond critical points (BCPs) of the Pb–O and Pb–S interactions in BTD obtained from QTAIM analysis: electron density ρ(r), its Laplacian ∇^2^ρ(r), Lagrangian kinetic energy density G(r), potential energy density V(r) and total energy density H(r) = G(r) + V(r).

BCP	Bond	ρ(r)	∇^2^ρ(r)	G(r)	V(r)	H(r)	|V(r)|/G(r)
27	Pb–O	0.0242	0.0617	0.0151	−0.0162	−0.0011	1.07
30	Pb–S	0.0336	0.0990	0.0239	−0.0275	−0.0036	1.15
33	Pb–O	0.0348	0.1021	0.0239	−0.0266	−0.0028	1.12
35	Pb–O	0.0268	0.0754	0.0178	−0.0190	−0.0012	1.07
37	Pb–O	0.0383	0.1308	0.0286	−0.0318	−0.0032	1.11
40	Pb–S	0.0364	0.0933	0.0144	−0.0176	−0.0032	1.22

**Table 4:** Calculated third-order nonlinear optical parameters from Z-scan experimental data.

Third-Order NLO Properties	Calculated Values
β ×10^−12^ (m/W)	3.0988
n_2_ × 10^−19^(m^2^/W)	2.7185
R_e_χ^(3)^ × 10^−13^ (esu)	0.8924
I_m_χ^(3)^ × 10^−13^ (esu)	0.4309
χ^(3)^ × 10^−13^ (esu)	0.9909
Optical limiting (J cm^−2^)	24.44

**Table 5:** Comparison of third-order nonlinear optical properties with reported thiourea-based crystals.

Crystal	χ^(3)^ (×10^−13^ esu)	Reference
Bisthiourea cadmium chloride (BTCC)	0.65	[28]
Bisthiourea zinc chloride (BTZC)	0.74	[15]
Bisthiourea zinc acetate (BTZA)	0.82	[12]
Tris(thiourea)zinc sulfate (ZTS)	0.91	[29]
Bisthiourea lead dinitrate (BTD)	0.9909	Present work

## Data Availability

All data generated or analyzed during this study are included in this manuscript.

## Abbreviations

BTD	bis(thiourea) lead dinitrate
FT-IR	Fourier transform infrared spectroscopy
TG-DTA	Thermogravimetric and differential thermal analysis
NLO	Nonlinear optical
UV-Vis DRS	Ultraviolet visible near-infrared diffuse reflectance spectroscopy
XRD	X-ray diffraction
CCDC	Cambridge Crystallographic Data Centre
DFT	Density functional theory
QTAIM	Quantum theory of atoms in molecules
BCP	Bond critical point
NCI	Non-covalent interaction
RDG	Reduced density gradient
MEP	Molecular electrostatic potential
ELF	Electron localisation function
LOL	Localised orbital locator
ALIE	Average local ionisation energy
IRI	Interaction region indicator
HOMO	Highest occupied molecular orbital
LUMO	Lowest unoccupied molecular orbital
DOS	Density of states
SCXRD	Single-crystal X-ray diffraction
PXRD	Powder X-ray diffraction
SEM	Scanning electron microscopy
EDS	Energy-dispersive X-ray spectroscopy
PL	Photoluminescence
RSA	Reverse saturable absorption
CA/ OA	Closed aperture/open aperture
